# Volumetric characteristics of idiopathic pulmonary fibrosis lungs: computational analyses of high-resolution computed tomography images of lung lobes

**DOI:** 10.1186/s12931-019-1189-5

**Published:** 2019-10-11

**Authors:** Bora Sul, Lucia Flors, Joanne Cassani, Michael J. Morris, Jaques Reifman, Talissa Altes, Anders Wallqvist

**Affiliations:** 1 Department of Defense Biotechnology High Performance Computing Software Applications Institute, Telemedicine and Advanced Technology Research Center, United States Army Medical Research and Development Command, FCMR-TT, 504 Scott Street, Fort Detrick, MD 21702-5012 USA; 2 The Henry M. Jackson Foundation for the Advancement of Military Medicine, Inc., Bethesda, 20817 MD USA; 3 Department of Radiology, University of Missouri, Columbia, 65211 MO USA; 4 Pulmonary/Critical Care, Brooke Army Medical Center, Joint Base San Antonio, Fort Sam Houston, 78234 TX USA

**Keywords:** Idiopathic pulmonary fibrosis, Quantitative HRCT analysis, Lobar flow distribution

## Abstract

**Background:**

Idiopathic pulmonary fibrosis (IPF) is a fatal lung disease involving progressive degeneration of lung capacity. Current diagnosis of IPF heavily relies on visual evaluation of fibrotic features in high-resolution computed tomography (HRCT) images of the lungs. Although the characteristics of this disease have been studied at the molecular and cellular levels, little is known about the mechanical characteristics of IPF lungs inferred from HRCT images. To this end, we performed a pilot study to investigate the radiographic and volumetric characteristics of lungs in subjects with IPF.

**Methods:**

We collected HRCT images of healthy (*N* = 13) and IPF (*N* = 9) lungs acquired at breath-holds after full inspiration (expanded state) and full expiration (contracted state). We performed statistical analyses on Hounsfield unit (HU) histograms, lobar volumes (*V*: lobe volume normalized by the lung volume), and lobar flows (*Q*: the difference in lobe volume divided by the difference in lung volume between the expanded and contracted states).

**Results:**

Parameters characterizing the HU histograms (i.e., mean, median, skewness, and kurtosis) significantly differed between healthy and IPF subjects, for all lobes in both expanded and contracted states. The distribution of *V* across lobes differed significantly between the groups in both states. The distribution of *Q* also differed significantly between the groups: *Q* values of the lower lobes for the IPF group were 33% (right) and 22% (left) smaller than those for the healthy group, consistent with the observation that radiographic scores were highest in the lower lung section in IPF. Notably, the root-mean-squared difference (RMSD) of *Q*, a measure of distance from the mean value of the healthy group, clearly distinguished the IPF subjects (RMSD of *Q* > 1.59) from the healthy group (RMSD of *Q* < 0.67).

**Conclusion:**

This study shows that lung volume and flow distribution change heterogeneously across the lung lobes of IPF subjects, with reduced capacity in the lower lobes. These volumetric changes may improve our understanding of the pathophysiology in IPF lungs.

## Background

Idiopathic pulmonary fibrosis (IPF) is a specific form of chronic and fibrosing interstitial pneumonia of unknown cause [1–3], and the most common and fatal among such interstitial lung disorders. Patients with IPF are characterized by progressively worsening dyspnea and have a median life expectancy of three to five years from the time of diagnosis [4].

Previous studies have identified molecular and cellular mechanisms [5–8] that are potentially associated with the onset and progression of IPF. More recently, a number of studies have demonstrated that the structural and mechanical characteristics of extracellular matrices and fibroblasts differ between normal and IPF tissues [9–11]. A particularly interesting study has shown that tissue stiffness is greater in fibroblasts from patients with IPF than those from normal subjects [12]. These studies suggest that changes in mechanical and structural characteristics of tissues could directly lead to reduced lung function in IPF subjects. Such changes could also affect lung function indirectly, as the behavior and structural characteristics of tissues could further change via positive feedback between stiffened tissues and their microenvironments [13, 14].

Several imaging studies, particularly those using high-resolution computed tomography (HRCT), have characterized abnormalities in the lungs of IPF patients [2, 15, 16]. These studies have shown that excessive and abnormal fibrosis in IPF lungs are manifested as numerous abnormal radiological features [17], including subpleural and basilar predominant reticulation, honeycombing, traction bronchiectasis and bronchiolectasis, and ground-glass opacification [15, 18]. In particular, these features have been invaluable in establishing HRCT imaging as a critical tool in diagnosing [15, 16, 19], staging, or monitoring the progression of IPF [20–22].

Despite these advances, diagnosis of IPF heavily relies on visual evaluation of the described fibrotic features in HRCT images, and assessment of the disease is subjective with no commonly accepted or standardized fibrotic score. In addition, we still have only a rudimentary understanding of how the structural changes in the lungs of IPF subjects affect their functional properties. Although increases in the stiffness of tissues are likely to reduce global lung function, whether such changes induce regional differences in the mechanical responses of lungs is not well understood. In addition, it is of interest to determine whether HRCT images can be used to detect such regional changes in lung function. To address these questions, here we investigated the radiological and volumetric characteristics of the lungs in healthy and IPF subjects. In particular, we compared mechanical quantities for individual lung lobes in both expanded and contracted lung states by quantitatively analyzing HRCT images.

## Methods

### Study subjects and pulmonary function tests

We recruited healthy and IPF subjects through advertisements in local hospitals near the University of Missouri, Columbia, MO. All IPF subjects enrolled in this study were diagnosed with the disease following the usual clinical work-up with imaging. None of the subjects had been diagnosed with emphysema at the time of enrollment. Although one subject (D9) showed mild signs of emphysema on imaging, this person had no clinical diagnosis of emphysema (e.g., FEV_1_ = 97% predicted). No other subject was diagnosed with COPD or emphysema, although many had shortness of breath.

Initially, we selected the IPF subjects based on their previous medical history, including the initial diagnosis and most recent prognosis of IPF based on HRCT imaging obtained prior to enrollment. Clinical diagnosis of IPF was based on clinical course, pulmonary function tests, and HRCT findings for all patients. According to the guidelines of the 2018 ATS [15] and the Fleischner Society [23], the UIP patterns were typical for six patients (D1, D2, D3, D6, D7, and D9), probable for two (D4 and D5), and indeterminate for two (D8 and one excluded from the study. We excluded this patient because the large amount of fibrosis in the lung led to high uncertainty in the segmentation of lung lobes.) Both patients with *indeterminate* UIP patterns and one (D4) with *probable* had a lung biopsy as part of the initial clinical work-up. The remaining patients were diagnosed based on their original clinical work-up and CT findings. Given that our investigation was a pilot study focused on imaging, we did not perform any additional testing (e.g., autoimmune panel testing) to confirm the original diagnosis of IPF or exclude alternative diagnoses. Healthy subjects had no history of pulmonary disease and had smoked less than 100 cigarettes in their lifetime. One healthy subject (H13) had smoked a few cigarettes over a 6-month period (less than 100 over a lifetime). None of the other healthy subjects had ever smoked. Four IPF subjects (D5, D6, D8, and D9) had a history of smoking (past or current) ranging from 15 to 20 years. One subject (D1) had smoked more than 40 years ago, but less than 100 cigarettes over a lifetime. The remaining IPF subjects had never smoked. All subjects were more than 18 years of age.

For each subject, we performed a full pulmonary function test (PFT; Vmax Encore PFT system, Vyaire Medical, Mettawa, IL), including measurements for lung volumes and diffusing capacity of carbon monoxide (DLCO). For the functional tests, we provided the values for the forced vital capacity (FVC), the forced expiration volume during one second (FEV_1_), the total lung capacity (TLC), and DLCO as *% predicted*, i.e., as a percentage of the normal reference value provided by the National Health and Nutritional Examination Survey III [24] and European Respiratory Society [25]. We defined the FEV_1_/FVC quantity conventionally as the ratio of the raw volumetric measurements (in liters) of FEV_1_ and FVC rather than as a ratio of %-predicted values.

For the physiology-based risk assessment of IPF, we calculated the gender-age-physiology (GAP) index [20], a composite score (range: 0–8) that considers scores for gender (G), age (A), and physiology (P). G is scored as 0 for women and 1 for men, and A as 0, 1, or 2 for age ranges ≤ 60, 61–65, or > 65, respectively. P denotes the sum of the subscores, P_FVC_ and P_DLCO_, which are associated with the FVC and DLCO, respectively. P_FVC_ is scored as 0, 1, or 2 if FVC is > 75% predicted, 50–75% predicted, or < 50% predicted, respectively. P_DLCO_ is scored as 0, 1, or 2 if DLCO is > 55% predicted, 36–55% predicted, or ≤ 35% predicted, respectively, or 3 if DLCO cannot be measured.

Table 1 shows a summary of the demographic data for healthy and IPF subjects, along with their full PFT results. The physiological variables of the IPF subjects indicated that their condition was mild. Their FVC and DLCO values were 85% predicted [standard deviation (SD) = 186% predicted] and 48% predicted (SD = 16% predicted), respectively. Similarly, the GAP index for the IPF subjects was 3.6 (SD = 1.4), which corresponds to IPF stage I, with a predicted 3-year mortality rate of 16% [20]. The TLC for the IPF subjects was 72% predicted (SD = 17% predicted). However, the FEV_1_ for the IPF subjects was 90% predicted (SD = 21% predicted). The FEV_1_/FVC for the IPF subjects was 0.81 (SD = 0.05). Although most healthy subjects had normal HRCT findings and lung function, some subjects showed mild deviation from the normal range. One subject (H12) showed normal HRCT findings but a FEV_1_ of 79% predicted. Four subjects (H5, H6, H7, and H11) showed normal pulmonary function (e.g., FEV_1_ = 84~129% predicted) but mild subpleural reticulation in the upper section (visual score < 10).

**Table 1:** Demographic characteristics of study subjects

Variable	Healthy	IPF	*p*-value
Sex	9 Women, 4 Men	4 Women, 5 Men	
Age (y)	61 (13)	71 (10)	0.107
Height (in)	66 (3)	65 (4)	0.950
Weight (lb)	150 (33)	168 (39)	0.549
GAP index^a^	1.3 (1.0)	3.6 (1.4)	0.003
FVC (% predicted)^b^	105 (14)	85 (16)	0.003
DLCO (% predicted)^b^	83 (12)	48 (16)	< 0.001
FEV_1_ (% predicted)^b^	102 (13)	90 (21)	0.231
FEV_1_/FVC^c^	0.75 (0.05)	0.81 (0.05)	0.010
TLC (% predicted)^b^	109 (18)	72 (17)	< 0.001

Mean (standard deviation) values of demographic variables and pulmonary function test results for the healthy (N = 13) and IPF (N = 9) groups. We used the t-test to evaluate the statistical significance of differences in the mean values between the two groups and made the family-wise Bonferroni correction to the *p*-values. Abbreviations: *DLCO* diffusing capacity of carbon monoxide, *FEV*_*1*_ forced expiratory volume in one second, *FVC* forced vital capacity, *GAP* gender-age-physiology, *IPF* idiopathic pulmonary fibrosis, *TLC* total lung capacity

^a^See Methods Section

^b^Entries for FVC, DLCO, FEV_1_, and TLC are expressed as a percentage of the normal reference value (% predicted)

^c^FEV_1_/FVC denotes the ratio of the raw volumetric measurements (in liters) of FEV_1_ and FVC

### HRCT imaging and radiological assessment

To compare volumetric changes in the lungs between expanded and contracted states, we collected thin-sliced (< 1 mm) HRCT images at two breath-holding conditions, one acquired after full inhalation and another after full exhalation. To optimize imaging resolution, we followed a previously reported protocol [26, 27] using a SOMATOM Definition Flash scanner (Siemens, Erlangen, Germany).

For quantitative assessment, thoracic radiologists (Drs. Altes and Flors, each with more than 14 years of experience) blinded to the patient’s clinical information visually scored HRCT images for the presence of subpleural reticulation, traction bronchiectasis and bronchiolectasis, ground-glass opacities, honeycombing, and emphysema, following a previously published guideline for scoring [28]. We scored variables to the nearest 5% in three zones in each lung as follows: *1*) upper zones: at or above the aortic arch, *2*) middle zone: between the aortic arch and inferior pulmonary veins, and *3*) lower zones: at or below the inferior pulmonary veins. Table 2 shows the visual scores averaged across subjects for the healthy and IPF groups. We provide the HRCT images for each subject in the Additional file 1: Figures S1 − S22).

**Table 2:** Mean (standard deviation) values of radiological scores for the healthy (N = 13) and IPF (N = 9) groups

Variable	Zone	Healthy	IPF	*p*-value
Subpleural reticulation	Upper	2.31 (3.88)	38 (27)	< 0.001
	Middle	0.00 (0.00)	51 (23)	< 0.001
	Lower	0.00 (0.00)	72 (25)	< 0.001
Traction	Upper	0.00 (0.00)	7 (10)	0.003
	Middle	0.00 (0.00)	24 (20)	< 0.001
	Lower	0.00 (0.00)	47 (32)	< 0.001
Ground-glass opacification	Upper	0.00 (0.00)	16 (10)	< 0.001
	Middle	0.00 (0.00)	30 (21)	< 0.001
	Lower	0.00 (0.00)	49 (26)	< 0.001
Honeycombing	Upper	0.00 (0.00)	3 (7)	0.082
	Middle	0.00 (0.00)	6 (11)	0.082
	Lower	0.00 (0.00)	13 (14)	0.001
Emphysema	Upper	0.00 (0.00)	2 (7)	0.247
	Middle	0.00 (0.00)	1 (3)	0.247
	Lower	0.00 (0.00)	0 (0)	N/A

We used the t-test to evaluate the statistical significance of differences in the meanvalues between the two groups and made the family-wise Bonferroni correction to the *p*-values. Abbreviation: *IPF* idiopathic pulmonary fibrosis

### Segmentation of lung images

For volumetric analyses, we performed lung and lobe segmentation of HRCT images. We conducted initial lung segmentation by setting a threshold range for the Hounsfield unit (HU) of each pixel in each HRCT image in the MIMICS software system (version 11, Materialise, Leuven, Belgium). For most images, a range of − 1024 to − 500 HU was sufficient to cover all lung regions [29]. To ascertain how the upper bound influenced the segmentation, we systematically adjusted the upper bound between − 700 and − 300 HU for a number of images, and found that the change in lung volume was less than 5%. Furthermore, to ensure that any difference between images of the expanded lung and contracted lung for an individual subject was not confounded by differences in the upper bound used to segment the images, we used the same threshold range to segment the images obtained during inhalation and exhalation conditions.

In most images of healthy lungs, the initial threshold-based segmentation was sufficient. However, for some images of IPF lungs, this segmentation procedure erroneously excluded regions with severe fibrosis from the lung domain. This was due to the difficulty of distinguishing between scarred lung tissue and its surrounding structures, for which the HU values were similar. In such cases, we manually included the left-out lung regions based on the shape and anatomy of the lung.

Next, we used the lung fissures to identify the lung lobes in the segmented lung regions. Although the major fissures were clearly identifiable for all lobes, in some cases, the minor fissure, which typically forms a border between the right upper and right middle lobes, did not exist or was not easily detectable. Because in such cases we had no systematic means of segmenting these lobes, we chose to group them together as part of the right upper lobe (RU) for all images. Thus, we segmented the lung regions into four lobes: the RU, right lower (RL), left lower (LL), and left upper (LU) lobes. Two radiologists (Drs. Altes and Flors) confirmed the identification of the lung fissures and the final segmentation results.

### Computation of HU distribution in lung HRCT images

We quantitatively analyzed the distributions of HU values of the pixels within the lung domain of the HRCT images by computing their normalized histograms (hereafter, HU histograms). We characterized each HU histogram by calculating its mean, median, skewness, and kurtosis of asymmetry of a distribution. Here skewness is defined as:

1 $$ \mathrm{skewness}=\frac{\overline{{\left(x-\overline{x}\right)}^3}}{\sigma_x^3} $$

where ${\sigma}_{\mathit{\mathsf{x}}}$ denotes the standard deviation of *x*. Kurtosis indicates the extent to which a distribution contains extreme outliers, and is defined as:

2 $$ \mathrm{kurtosis}=\frac{\overline{{\left(x-\overline{x}\right)}^4}}{\sigma_x^4} $$

Here, *x* denotes the HU value of a pixel and $\overline{x}$ represents its mean across all pixels in the lung domain [30].

### Computation of anatomical and mechanical characteristics of lung lobes

We quantified the anatomical and mechanical characteristics of the lung lobes using the following quantities:

**Lobar volume (*V*):** the volume of a lung lobe normalized by the volume of the whole lung.

**Lobar flow (*Q*):** the difference in the volume of a lobe between the expanded and contracted states, normalized by the difference in the lung volume between the two states. The difference in the lung volume corresponds to the total amount of air breathed. Therefore, *Q* represents the amount of air breathed at each lobe as a fraction of this total. For each lobe *i* (*i* = RU, RL, LL, and LU), *Q*_*i*_ is defined as:

3 $$ {Q}_i=\frac{v_{e,i}-{v}_{c,i}}{\sum_{i=1}^4\left({v}_{e,i}-{v}_{c,i}\right)} $$

where *v*_*e,i*_ and *v*_*c,i*_ denote the volumes of lobe *i* measured in the expanded and contracted states, respectively.

**Lobar strain (*S*):** we defined the volumetric strain of each lobe *i* (*i* = RU, RL, LL, and LU) as the difference in lobe volume between the expanded and contracted states, normalized by the lobe volume in the expanded state:

4 $$ {S}_i=\frac{v_{e,i}-{v}_{c,i}}{v_{e,i}} $$

**Root-mean-squared difference (RMSD) of a quantity** ***X*:** for each individual, this measure indicates the distance between the value of *X* for the individual and the mean value of *X* for the healthy group (${X}_i^h$), normalized by the standard deviation of *X* for the healthy group (${\sigma}_i^h$). This is defined as:

5 $$ \mathrm{RMSD}\kern0.5em \mathrm{of}\kern0.5em \mathrm{X}=\kern0.5em {\sum}_{\mathrm{i}=1}^4\frac{\sqrt{{\left({X}_i-{X}_i^h\right)}^2}}{4{\sigma}_i^h} $$

where lobe *i* = RU, RL, LL, and LU.

We assessed the robustness of the RMSD of a particular quantity using the leave-one-out cross validation procedure [31], by computing the mean and standard deviation of the RMSD for 12 healthy subjects 13 times, leaving out a different healthy subject each time.

### Statistical analyses

We used the t-test to assess whether there were statistically significant differences between the healthy and IPF groups. We made corrections for *p*-values using the Bonferroni method to control for the family-wise error rate [32]. For each quantity of comparison, we used the Kolmogorov–Smirnov test to confirm that the data were normally distributed [32].

## Results

### Radiological characteristics of idiopathic pulmonary fibrosis lungs

All IPF lungs showed HRCT features characteristic of usual interstitial pneumonia (UIP), with subpleural and basilar predominant reticulation, traction bronchiectasis, and honeycombing. Table 2 shows the group average and SD of the radiological scores. For the IPF subjects, the group-average radiological scores for subpleural reticulation, traction bronchiectasis/bronchiolectasis, ground-glass opacification, and honeycombing were highest in the lower section of the lung and lowest in the upper section. Signs of emphysema were present only in one subject, whose scores were 20 and 10 at the upper and middle sections of the lung, respectively. The healthy subjects did not show any abnormalities, except for two subjects, who each showed minimal biapical scarring (radiological score = 5).

### Hounsfield units for healthy and idiopathic pulmonary fibrosis lungs

**Fig. 1:**
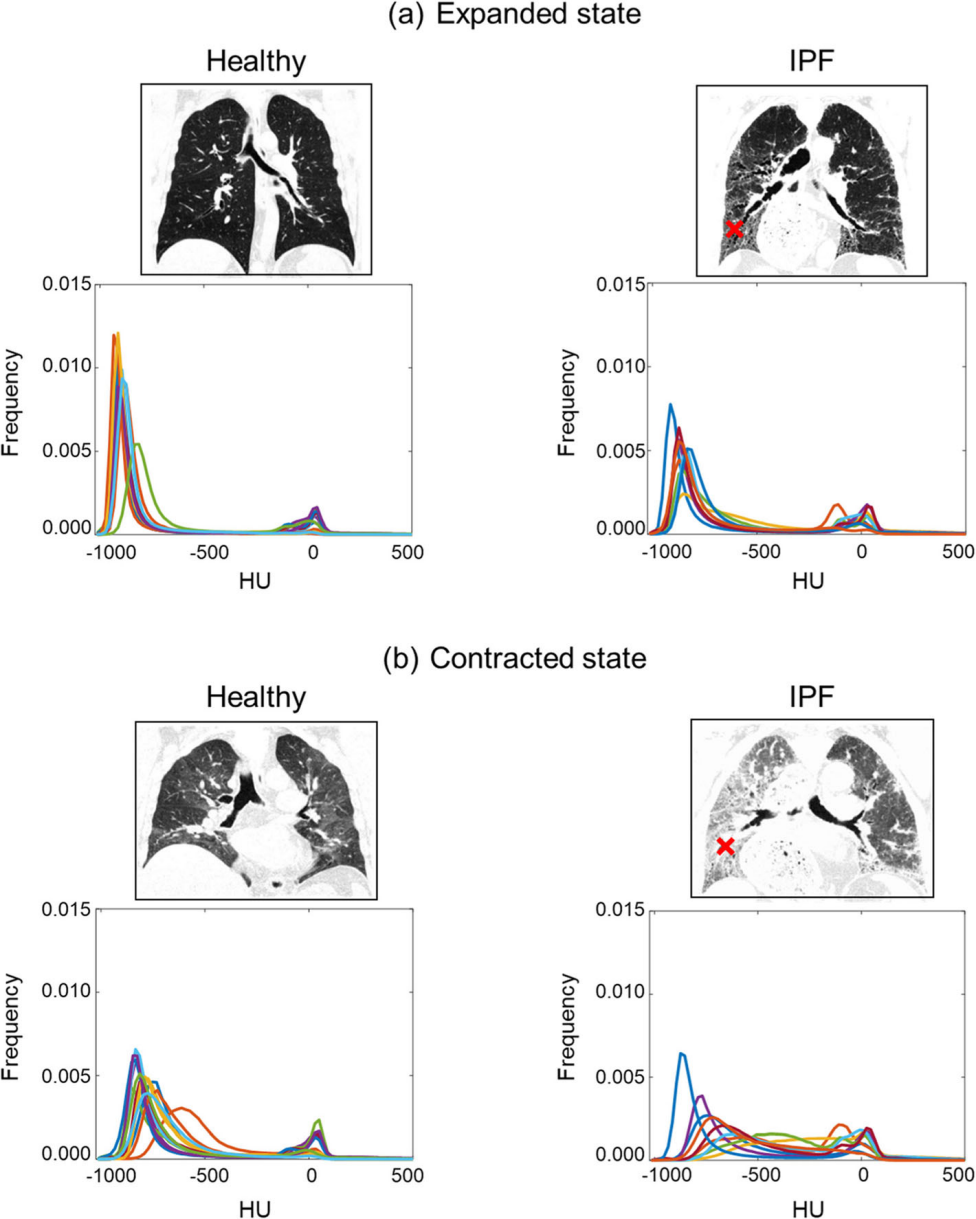
High-resolution computed tomography (HRCT) images of a healthy subject and an idiopathic pulmonary fibrosis (IPF) subject and histograms of Hounsfield units (HU), normalized by the total number of occurrences, for HRCT images of healthy (*N* = 13; left panel) and IPF (*N* = 9; right panel) subjects. For each subject, we acquired HRCT images at two breath-holds, one after full inspiration (Expanded; **a**) and another after full expiration (Contracted; **b**). Fibrotic regions are cross-marked (red) in the HRCT images of the IPF subject. Each color in a histogram represents an individual subject

To determine the radiographic differences in lung HRCT images between the healthy and IPF subjects computationally, we examined the HU values, which are associated with the radiological density of lung substances for the whole lung region. HU values for fibrotic lung regions (Fig. 1, red cross-marks in the right panel) were typically within a range between − 200 and − 600, as observed in previous studies [33, 34].

Figure 1 shows HU histograms in the expanded (Fig. 1a) and contracted (Fig. 1b) lungs for the healthy and IPF subjects. Because we did not exclude pulmonary vasculature, for all subjects, the HU histogram was bimodal with peaks near HU values of − 1000 (*left peak*) and 0 (*right peak*), which correspond to those for air and water, respectively [29]. However, the width and asymmetry of the *left peak* differed between the healthy and IPF subjects and between the expanded and contracted states. As expected for both IPF and healthy subjects, the lung density increased in the contracted state because of expiration of air from the lung. This is indicated by the reduced height and rightward shift of the left peak for both healthy and IPF subjects (Fig. 1). The mean and median HU values averaged across subjects for each group were higher in the contracted state (i.e., expiration) for both healthy and IPF subjects, reflecting the rightward shift of the histogram (Table 3). The skewness (Eq. 1), which quantifies the asymmetry of the histogram, was lower in the contracted state (Table 3). Similarly, kurtosis (Eq. 2), which we used to measure the extent to which the histogram included extreme outliers, was smaller in the contracted state than in the expanded state for both healthy and IPF subjects, although the difference between states was greater for the healthy subjects (Table 3).

**Table 3:** Mean (standard deviation) values of HU for the healthy (N = 13) and IPF (N = 9) groups

Variable		Healthy	IPF	*p*-value
Expanded state	Mean	− 739 (100)	− 596 (69)	0.003
	Median	− 869 (48)	− 750 (64)	< 0.001
	Kurtosis	8.93 (5.74)	3.54 (1.12)	0.018
	Skewness	2.33 (0.97)	1.18 (0.34)	0.006
Contracted state	Mean	− 578 (60)	− 382 (133)	0.002
	Median	− 690 (55)	− 429 (184)	0.002
	Kurtosis	5.10 (1.99)	3.05 (1.01)	0.007
	Skewness	1.49 (0.39)	0.56 (0.50)	0.001

We used the t-test to evaluate the statistical significance of differences in the mean values between the two groups and made the family-wise Bonferroni correction to the *p*-values. Abbreviations: *HU* Hounsfield unit, *IPF* idiopathic pulmonary fibrosis

Overall HU values for the IPF lungs were higher than those for healthy lungs in both expanded and contracted states. In both states, the *left peak* was broader and shifted further rightward for IPF subjects than they were for healthy subjects (Fig. 1). For all variables in both expanded and contracted states, group differences between the healthy and IPF subjects were statistically significant (*p* < 0.05) (Table 3). Moreover, the differences in the mean, median, and skewness between the IPF and healthy subjects were greater in the contracted state than in the expanded state. Specifically, average HU mean values for the IPF subjects in the expanded and contracted states were 19 and 34% higher, respectively, than those for the healthy subjects (Table 3). Average skewness values for IPF subjects in the expanded and contracted states were 49 and 62% lower, respectively, than those for the healthy subjects (Table 3). Finally, average kurtosis values for IPF subjects in the expanded and contracted states were 60 and 40% lower, respectively, than those for the healthy subjects (Table 3).

**Fig. 2:**
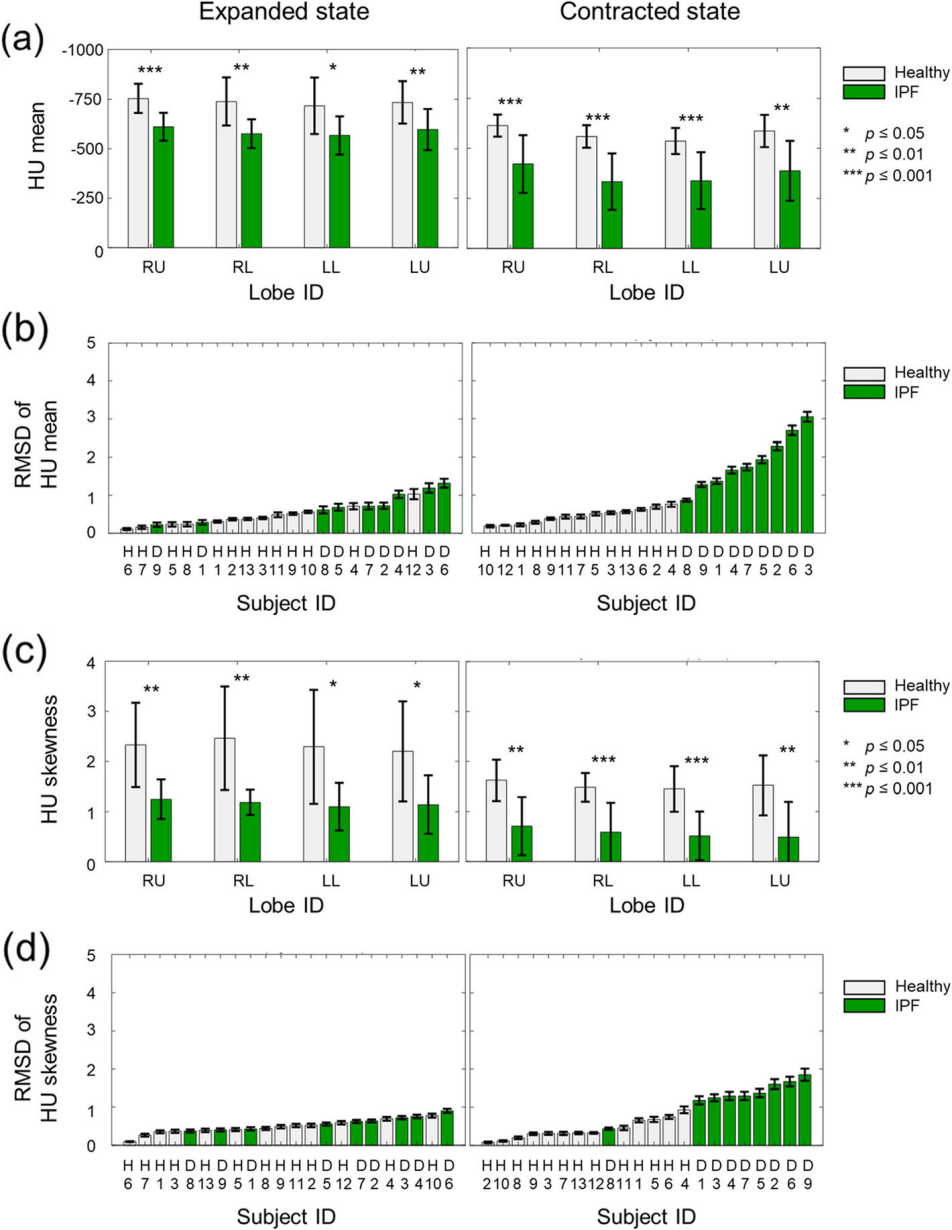
Mean and skewness values of Hounsfield units (HU) of lungs in their expanded (left panel) and contracted (right panel) states for healthy (*N* = 13) and idiopathic pulmonary fibrosis (IPF) subjects (*N* = 9). We computed these values from the HU histograms shown in Fig. 1. (**a**) HU means averaged for the healthy and IPF groups. (**b**) Individual root-mean-squared difference (RMSD) values of the HU mean. (**c**) HU skewness values averaged for the healthy and IPF groups. (**d**) Individual RMSD values of HU skewness. In **a** and **c**, the error bars denote one standard deviation and the asterisks indicate different levels of statistical significance for differences in the mean values between the two groups. In **b** and **d**, the error bars denote one standard deviation computed from 13 iterative leave-one-out validations (See Methods Section). RU denotes the right upper and right middle lobes combined. RL, LL, and LU denote the right lower, left lower, and left upper lobes, respectively

Figure 2 shows the lobar characteristics averaged across subjects for each group, measured by the mean (Fig. 2a and b) and skewness (Fig. 2c and d) of the HU histogram in the four lobes in the expanded (left panels) and contracted (right panels) states. In both states, the HU mean, and hence lung density, was significantly larger (i.e., less negative) for the IPF subjects (Fig. 2a). Differences in the HU mean between the healthy and IPF subjects were slightly greater in the contracted state than in the expanded state (Fig. 2a): in the expanded state, HU means for the IPF subjects were roughly 20% greater than those for the healthy subjects in all lobes, whereas in the contracted state, they were 30 to 40% greater.

Similarly, skewness differed significantly between the healthy and IPF subjects for all lobes in both expanded and contracted states (Fig. 2c); in the expanded state, the skewness for IPF subjects was roughly 50% smaller than that for the healthy subjects, while in the contracted state, it was roughly between 56 and 66% smaller than that for the healthy group.

Figure 2b and d show RMSD values, which quantify the distance from the average of the healthy group (Eq. 5), of the lobar mean and skewness for each individual, respectively. The RMSD of the lobar mean clearly separated the healthy and IPF subjects in the contracted state (Fig. 2b, right panel), but not in the expanded state (Fig. 2b, left panel). Similarly, the RMSD of the lobar skewness separated the healthy subjects and IPF subjects more clearly in the contracted state (Fig. 2d, right panel) than it did in the expanded state (Fig. 2d, left panel). The RMSD of the lobar skewness for one IPF subject (D9) fell within the range of the healthy group (Fig. 2d, right panel).

### Distribution of lobar volumes differs between healthy and idiopathic pulmonary fibrosis lungs

**Fig. 3:**
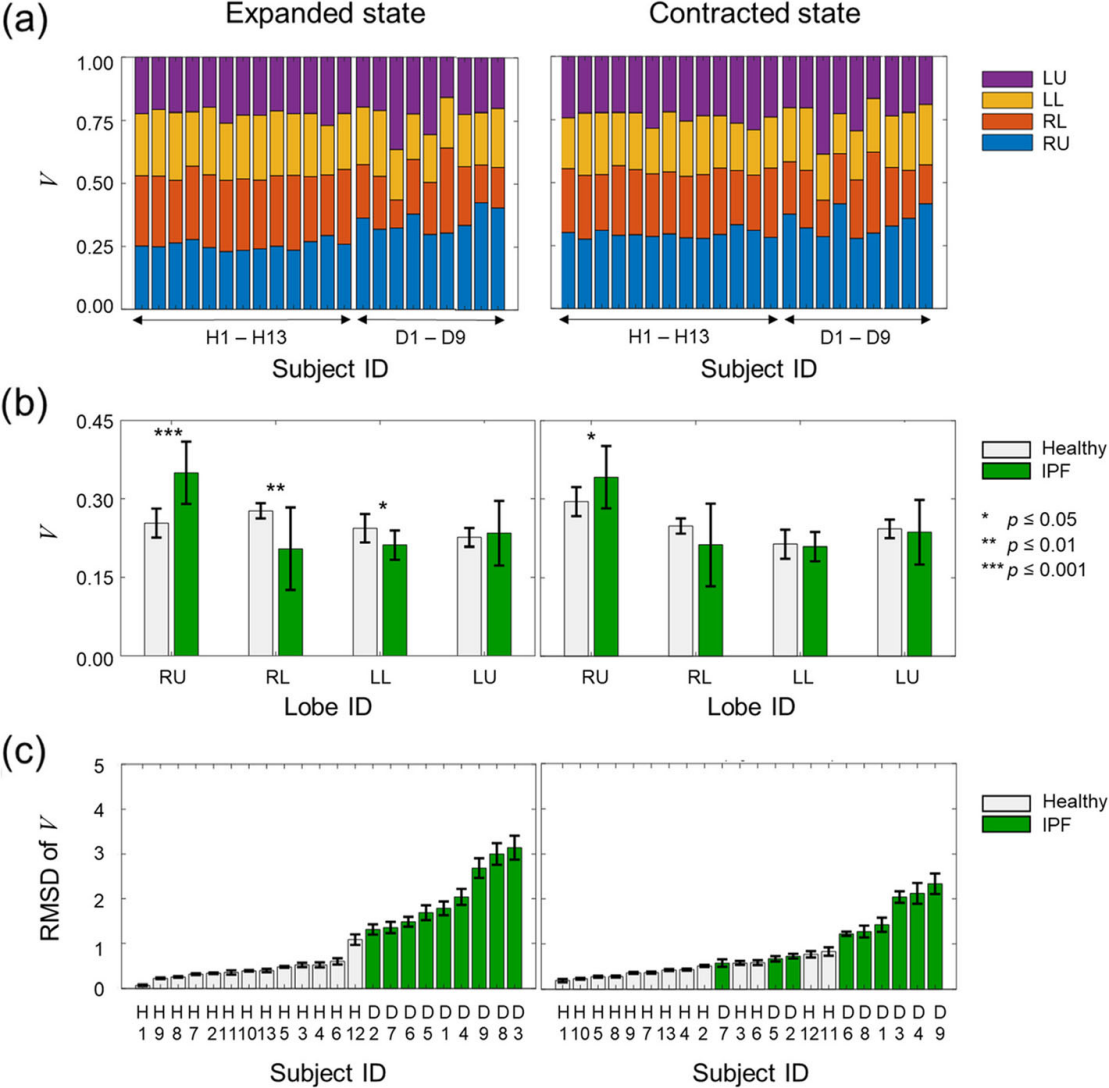
The lobar volume *V*, defined as the lobe volume normalized by the lung volume, for the healthy (*N* = 13) and idiopathic pulmonary fibrosis (IPF) subjects (*N* = 9). We measured *V* at two breath-holds, one obtained after full inspiration (expanded state) and another after full expiration (contracted state). (**a**) Individual *V* values for healthy (H1-H13) and IPF subjects (D1-D9). (**b**) Mean values of *V* for the healthy and IPF groups. The error bars denote one standard deviation and the asterisks indicate different levels of statistical significance for differences in the mean values between the two groups. (**c**) Individual root-mean-squared difference (RMSD) values of *V* (See Eq. 5 in the Methods Section). The error bars denote one standard deviation computed from 13 iterative leave-one-out validations (See Methods Section). RU denotes the right upper and right middle lobes combined. RL, LL, and LU denote the right lower, left lower, and left upper lobes, respectively

Figure 3 shows the lobe volume normalized by the lung volume (lobar volume *V*, Eq. 3) in the expanded (left panel) and contracted (right panel) states. For each lobe, the value of *V* averaged over the subjects in each group differed between the healthy and IPF groups in both the expanded and contracted states (Fig. 3b). For the IPF group, *V* for RU (34% in both expanded and contracted states) was higher than that for all other lobes, which showed similar values (ranging from 21 to 23%) in both expanded and contracted states. In contrast, for the healthy subjects, *V* in the expanded state was greatest for RL (28%). In the contracted state, it was greatest for RU (30%), differing significantly (*p* < 0.001) from all other lobes where it ranged from 21 to 25%.

Figure 3c shows the RMSD of *V* for each subject. The RMSD clearly separated the healthy and IPF groups in the expanded state (Fig. 3c, left). Although the RMSD did not separate the healthy and IPF groups as well in the contracted state (Fig. 3c), it was still able to discriminate most of the subjects in the two groups.

### Distribution of lobar flow differs between healthy and idiopathic pulmonary fibrosis lungs

**Fig. 4:**
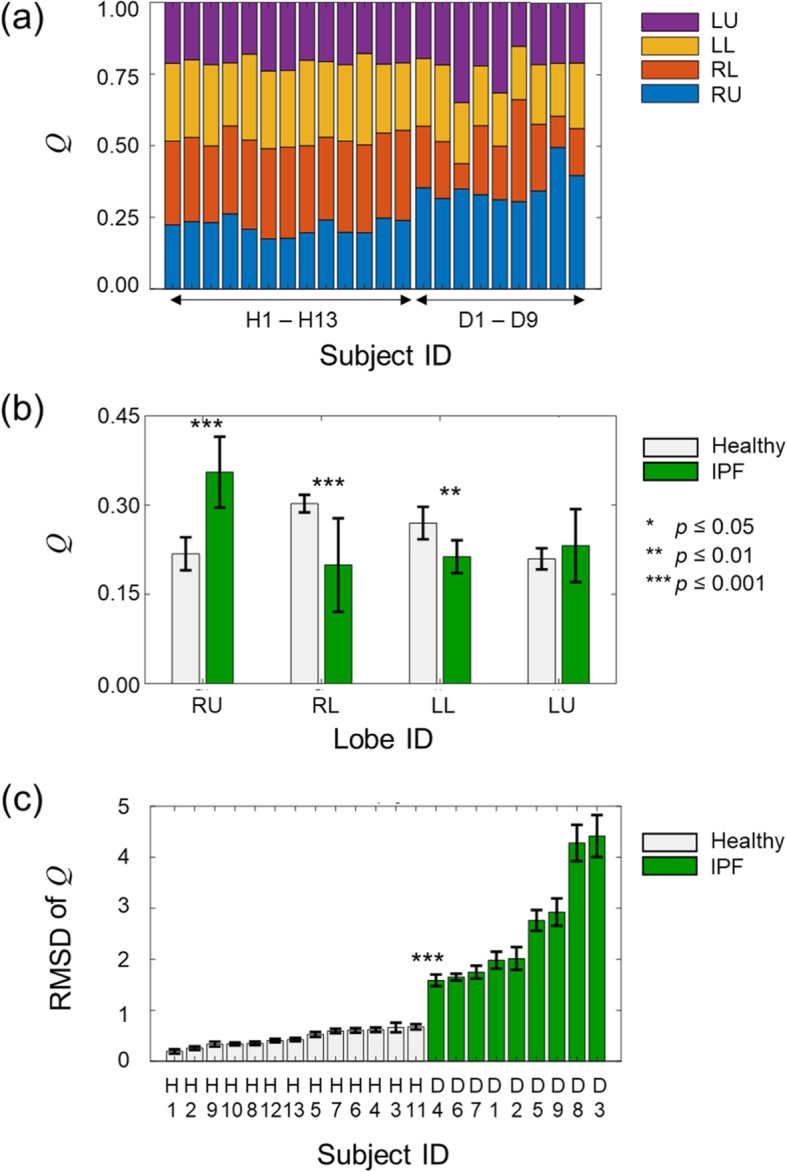
The lobar flow (*Q*) for the healthy (*N* = 13) and idiopathic pulmonary fibrosis (IPF) subjects (*N* = 9). *Q* is defined as the difference in lobe volume normalized by the difference in lung volume between the expanded and contracted states (See Eq. 3 in the Methods Section). (**a**) *Q* values of healthy (H1-H13) and IPF subjects (D1-D9). (**b**) Mean values of *Q* for the healthy and IPF groups. The error bars denote one standard deviation and the asterisks indicate different levels of statistical significance for differences in the mean values between the two groups. (**c**) The root-mean-squared difference (RMSD) of *Q* for individual subjects (See Eq. 3 in the Methods Section). The error bars denote one standard deviation computed from 13 iterative leave-one-out validations (See Methods Section). RU denotes the right upper and right middle lobes combined. RL, LL, and LU denote the right lower, left lower, and left upper lobes, respectively

Figure 4 shows the lobar flow *Q* (Eq. 3), defined as the difference in lobe volume divided by difference in lung volume between the expanded and contracted states, for the healthy and IPF subjects. The *Q* averaged across subjects for each group was 22% (RU), 30% (RL), 27% (LL), and 21% (LU) for the healthy subjects, and 36% (RU), 20% (RL), 21% (LL), and 23% (LU) for the IPF subjects. As such, the values of *Q* differed significantly between the healthy and IPF groups in every lobe except LU. In addition, the distribution of *Q* across the lobes differed between the two groups. For the healthy subjects, *Q* values for the lower lobes (30% for RL and 27% for LL) were larger than those for the upper lobes (22% for RU and 21% for LU). In contrast, for the IPF subjects, *Q* values for RU (36%) were significantly larger (*p* < 0.001) than those for all other lobes, which were comparable (20 to 23%).

**Fig. 5:**
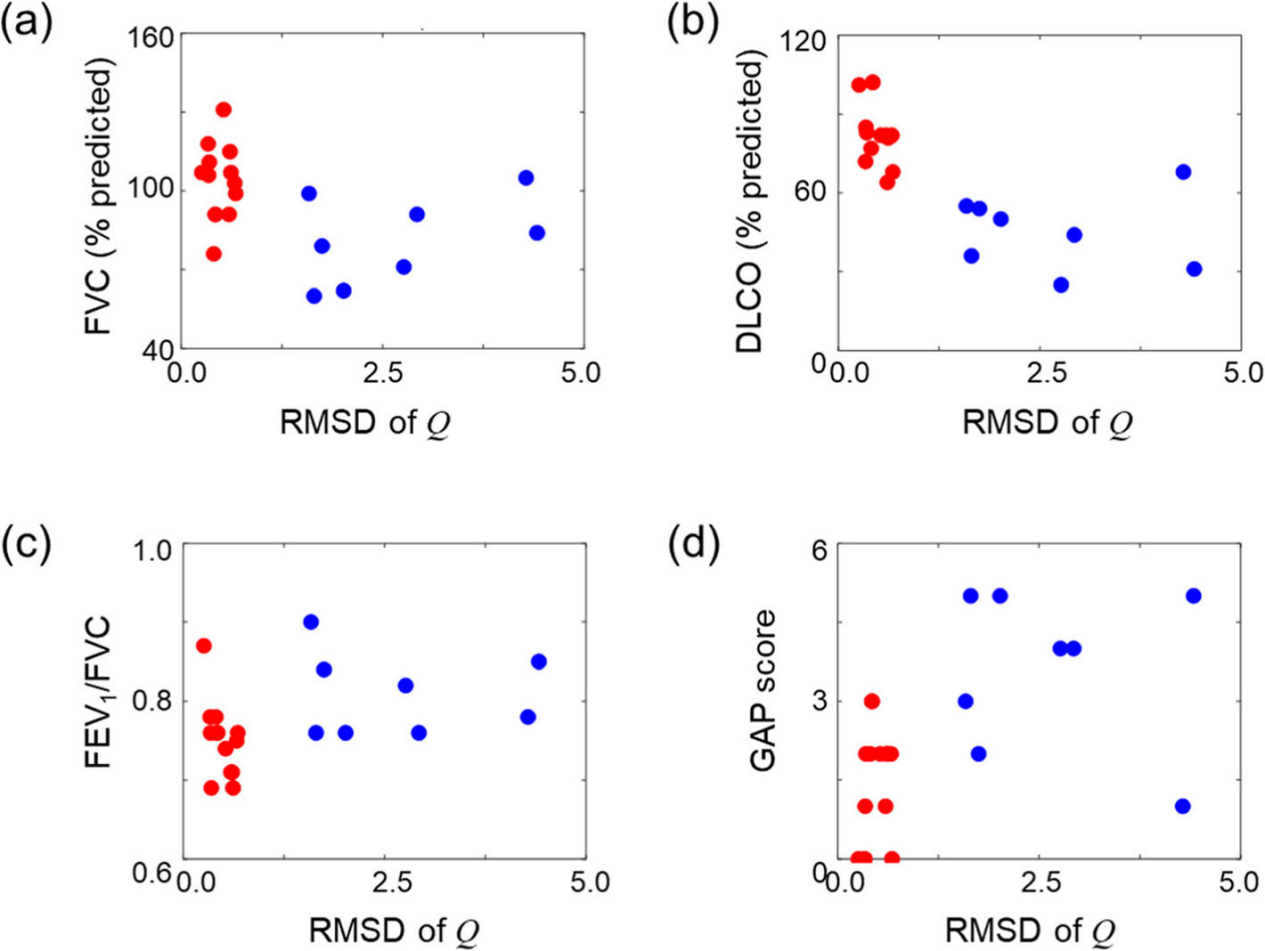
The lobar flow (Q) root-mean-squared difference (RMSD) plotted as a function of the (**a**) forced vital capacity (FVC), (**b**) diffusing capacity of carbon monoxide (DLCO), (**c**) ratio between the forced expiratory volume in one second (FEV1) and FVC, and (**d**) gender-age-physiology (GAP) index (See Methods Section). The unit of % predicted represents the value of the quantity expressed as a percentage of the normal reference value (See Methods Section). Values for healthy (*N* = 13) and idiopathic pulmonary fibrosis (IPF) subjects (*N* = 9) are shown as red and blue dots, respectively.

Importantly, the *Q* RMSD clearly separated the healthy subjects from the IPF subjects (Fig. 4c). The largest *Q* RMSD for the healthy group (0.66 for H11) differed significantly (*p* < 0.001) from the lowest value (1.59 for D4) for the IPF group. The *Q* RMSD averaged across subjects was 0.46 (SD = 0.16) for the healthy group and 2.56 (SD = 1.08) for the IPF group (Table 4). The *Q* RMSD clearly grouped the healthy subjects and the IPF subjects, in agreement with their physiological results (Fig. 5).

**Table 4:** Mean (standard deviation) values of volumetric variables for the healthy (N = 13) and IPF (N = 9) groups

Variable	Healthy	IPF	*p*-value
Lung volume in the expanded state, *v*_*e*_ (L)	5.56 (0.90)	4.07 (0.98)	0.003
Lung volume in the contracted state, *v*_*c*_ (L)	2.46 (0.56)	1.82 (0.30)	0.009
Lung volume difference, *v*_*e*_ -* v*_*c*_ (L)	3.10 (0.91)	2.24 (0.71)	0.021
Lung strain, (*v*_*e*_ - *v*_*c*_)/*v*_*e*_	2.20 (0.46)	2.15 (0.23)	0.592
RMSD^a^ of			
Lobar volume in the expanded state	0.43 (0.24)	2.04 (0.70)	< 0.001
Lobar volume in the contracted state	0.44 (0.20)	1.36 (0.66)	< 0.001
Lobar flow	0.46 (0.16)	2.56 (1.08)	< 0.001
Lobar strain	0.39 (0.30)	0.37 (0.15)	0.395

We used the t-test to evaluate the statistical significance of differences in the mean values between the two groups and made the family-wise Bonferroni correction to the *p*-values. Abbreviations: *IPF* idiopathic pulmonary fibrosis, *L* liter, *v*_*c*_, lung volume in the contracted state, *v*_*e*_, lung volume in the expanded state, *RMSD* root-mean-squared difference

^a^See Methods Section, Eq. 5

We computed the lobar strain *S* (Eq. 4), defined as the difference in lobe volume between the expanded and contracted states divided by the lobe volume in the expanded state (See Additional file 1: Figure S23). This metric indicates the volumetric strain of each lobe during lung contraction. *S* did not significantly differ between the healthy and IPF subjects for any lobe (Additional file 1: Figure S23b). Not surprisingly, the *S* RMSD could not separate the healthy subjects from the IPF subjects (Additional file 1: Figure S23c).

## Discussion

In this study, we quantitatively investigated the radiological characteristics and volumetric changes of each lobe for healthy and IPF subjects. The parameters of the HU histogram profiles, such as the mean, median, skewness, and kurtosis, significantly differed between the healthy and IPF subjects in all lobes, in agreement with previous studies of individual lung lobes [22] and the whole lung [35] in IPF. Nonetheless, most of these parameters and their RMSDs did not clearly separate healthy subjects from those with IPF. The one exception was the RMSD of HU mean in the contracted state, which separated the two groups (Fig. 2b, right panel).

The difference in HU histogram profiles between the healthy and IPF subjects may indicate an increase of fibrotic areas in lungs with IPF [17]. Because fibroblasts from patients with IPF are stiffer than normal lung tissue [12], we expected that the mechanical properties of the whole lung would differ between IPF and healthy subjects. However, whether regional differences in lung function would be manifested in HRCT images was not obvious.

Notably, our study showed that quantities associated with the dynamic responses of lungs differed lobe-wise between the healthy and IPF subjects. In particular, the pattern of volume distribution among the lung lobes differed between the healthy and IPF subjects, especially in the expanded state (Fig. 3b, left panel). For the IPF subjects, RU occupied by far the largest portion of the lung volume, whereas for the healthy subjects, RL occupied the greatest proportion. It is unclear why the pattern of lobar volume distribution between the healthy and IPF subjects would be more pronounced in the expanded state. Given the hysteresis of the pressure-volume loop [36], we might expect the lungs to show different dynamic characteristics during expansion and contraction. However, it is currently unknown whether fibrosis or other pathological features in IPF lungs affect expansion and contraction differently.

The presence of fibrotic tissue in the lung should increase lung stiffness in IPF subjects. However, one cannot measure lung stiffness in vivo without knowing the pressure applied to the lung during lung expansion or contraction. Nonetheless, under conditions in which the pressure in the lung is approximately uniform across the lung lobes, we can use the inverse of the lobar flow as an index of the stiffness of a lung lobe relative to the lung stiffness for each subject.

Interestingly, the distribution of lobar flows, associated with the lobar stiffness, differed between the healthy and IPF subjects (Fig. 4b). Specifically, the lobar flows of the lower lobes for the IPF subjects were smaller than those for the healthy subjects (Fig. 4b, RL and LL), whereas the lobar flow of the right upper lobe for the IPF subjects was higher than that for the healthy subjects (Fig. 4b, RU). This may indicate that lung stiffness for the IPF subjects in the lower lobes was higher than that in the upper lobes. In fact, visually determined radiological scores for all IPF features were highest in the lower section of the lung (Table 2), consistent with previous studies that show the greatest amount of fibrotic tissue in the lower sections [15, 18]. Alternatively, fibrosis might not only increase the lung stiffness in the lower lobes, but also alter active mechanisms involved in lung contraction. If so, the pressure distribution across the lobes might differ between the IPF and healthy subjects, which could affect the extent of lobar contraction for the IPF subjects. Further studies to identify such mechanisms should improve our understanding of the phenomena that alter the pattern of lobar contraction in IPF subjects.

The altered distribution of lobar flows in IPF implies both global and local changes in airflow patterns. Previous studies showed that airflow patterns in individual airway branches are sensitive to the distribution of lobar flows [27, 37]. In particular, due to an increased disparity in lobar flows between lung lobes for IPF subjects (Fig. 4b), the stress on the airway branches belonging to RU, where the flow rate is higher than other lobes, will be much higher than the stress on other airway branches. This may, in turn, induce mechanical fatigue and lead to airway damage [37].

It is noteworthy that some quantities we investigated not only differed between the healthy and IPF subjects in terms of their group averages, but also their individual (leave-one-out) RMSD values. Specifically, the RMSD of the HU mean in the contracted state (Fig. 2b, right panel), lobar volume in the expanded state (Fig. 3c, left panel), and lobar flow (Fig. 4c) clearly separated the healthy group from the IPF group. In particular, the RMSD of lobar flow achieved the clearest separation between the two groups, as the lower bound of the IPF group (1.59 for subject D4 in Fig. 4c) was 1.4 times the upper bound of the healthy group (0.67 for subject H11 in Fig. 4c). These findings suggest that the RMSD of lobar flow could be used to differentiate IPF subjects from healthy ones.

Our study was intended as a pilot study involving a small number of subjects (13 healthy and 9 IPF subjects). Therefore, the generalizability of these results to the broader population may be limited. Hence, to test the findings identified herein, we will need to conduct a prospective investigation involving a large number of healthy and IPF subjects. In addition, it is unclear whether the differences in the HU histogram profiles and mechanical properties we observed are specific to IPF. Answering this question will require follow-up studies comparing subjects with IPF to subjects with other types of lung diseases. For example, recent reports show that comorbidity of IPF and emphysema affects disease prognosis and mortality prediction [38]. Thus, it will be of interest to compare lobar flow patterns between patients with IPF only and those with comorbidity of IPF and emphysema.

The anatomical and functional characteristics measured in our study could potentially be useful for diagnosing and characterizing IPF. Currently, it remains a challenge to accurately diagnose IPF and stage its progression given the lack of established metrics to assess disease severity. Although HRCT plays a crucial role in IPF diagnosis, an understanding of the underlying pathological changes associated with the images is lacking. Our study shows a significant increase in the stiffness of the lower lobes in IPF lungs, demonstrating that HRCT imaging can be used to probe functional impairment in different lung regions. It will be of particular interest to use HRCT imaging to investigate lung stiffness associated with regional changes in lung tissues [e.g., proliferation of fibroblasts [39]], as they are correlated with UIP patterns in thin-section CT images of IPF lungs [17]. Concurrent scoring of functional changes and staging of disease state would aid in understanding the pathogenesis and progression of IPF.

## Conclusions

We performed a pilot study to investigate the structural and functional changes in the lungs of patients with IPF by quantitatively analyzing HRCT images of the lungs of healthy subjects and subjects with IPF. Hounsfield unit histograms, proportions of lobar volume, and lobar flow derived from the lung images differed significantly between healthy and IPF subjects, suggesting that both structural and functional changes in the lung occur in IPF. Interestingly, of the several statistical measures we explored for their ability to classify diseased and healthy subjects, the RMSD of lobar flow clearly separated the two subject groups. This quantity could potentially be useful in distinguishing IPF from healthy conditions.

## Supplementary information

**Additional file 1:** Figures S1−S13 show high-resolution computed tomography (HRCT) images and Hounsfield (HU) histograms of healthy subjects H1 through H13. Figures S14−S22 show HRCT images and HU histograms of idiopathic pulmonary fibrosis (IPF) subjects D1 through D9. Figure S23 shows the strain of the lung lobes for the healthy (N = 13) and IPF subjects (N = 9).

## Data Availability

The datasets generated and analyzed during the current study are not publicly available due to limited consent from study participants but are available from the corresponding author on reasonable request and regulatory approval.

## Abbreviations

% predicted: percentage of the normal reference value
COPD: Chronic obstructive pulmonary disease
DLCO: Diffusing capacity of carbon monoxide
FEV_1_: Forced expiratory volume in one second
FVC: Forced vital capacity
HRCT: high-resolution computed tomography
HU: Hounsfield units
IPF: Idiopathic pulmonary fibrosis
PFT: Pulmonary function testing
Q: lobar flow
RMSD: Root-mean-squared difference
S: lobar strain
SD: Standard deviation
TLC: Total lung capacity
UIP: Usual interstitial pneumonia
V: lobar volume
*v*_*c*_: lung volume in the contracted state
*v*_*e*_: lung volume in the expanded state
