# Supplementary material for: Volumetric characteristics of idiopathic pulmonary fibrosis lungs: computational analyses of high-resolution computed tomography images of lung lobes
Source: Respir Res. 2019 Oct 11;20:216. doi: 10.1186/s12931-019-1189-5 (PMC6787976; doi:10.1186/s12931-019-1189-5)
Supplement: Supplementary file 1 — Additional file 1: Figures S1−S13 show high-resolution computed tomography (HRCT) images and Hounsfield (HU) histograms of healthy subjects H1 through H13. Figures S14−S22 show HRCT images and HU histograms of idiopathic pulmonary fibrosis (IPF) subjects D1 through D9. Figure S23 shows the strain of the lung lobes for the healthy (N = 13) and IPF subjects (N = 9). [file 12931_2019_1189_MOESM1_ESM.pdf]

SUPPLEMENTARY MATERIAL

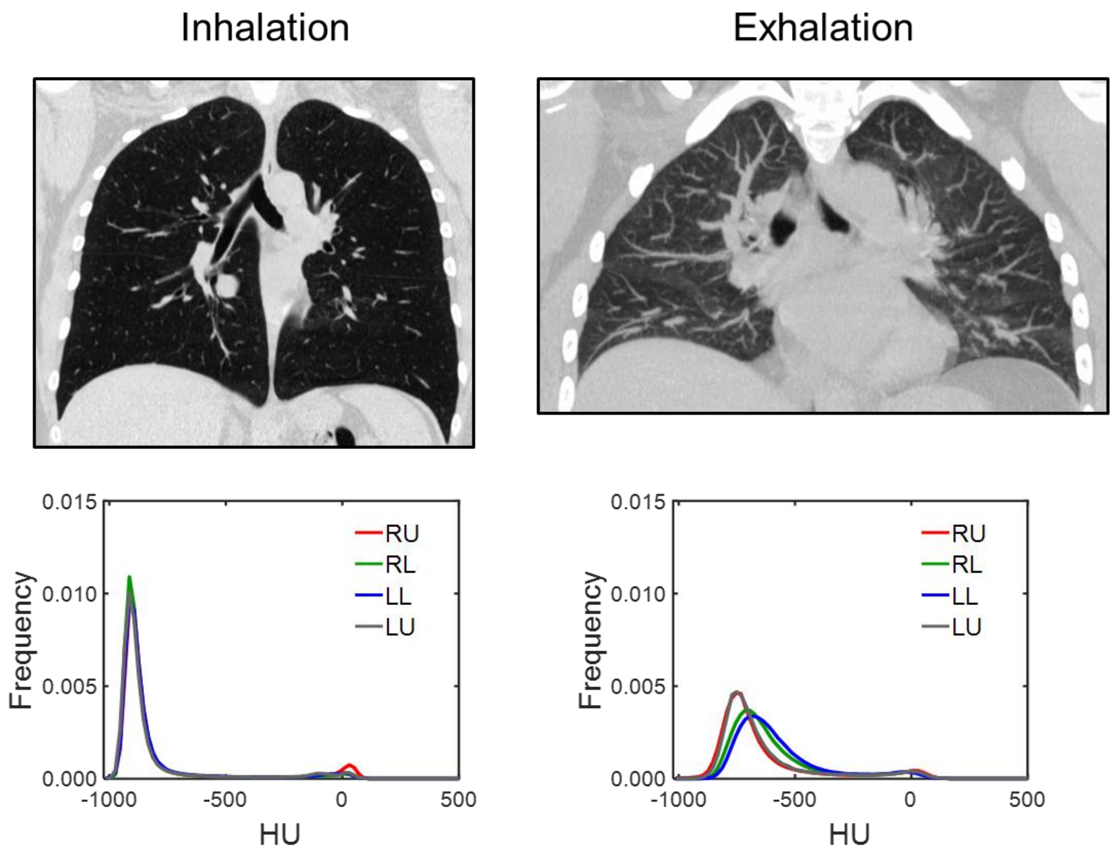

Figure S1

High-resolution computed tomography images and Hounsfield unit (HU) histograms of healthy subject H1. RU denotes the right upper and right middle lobes combined. RL, LL, and LU denote the right lower, left lower, and left upper lobes, respectively.

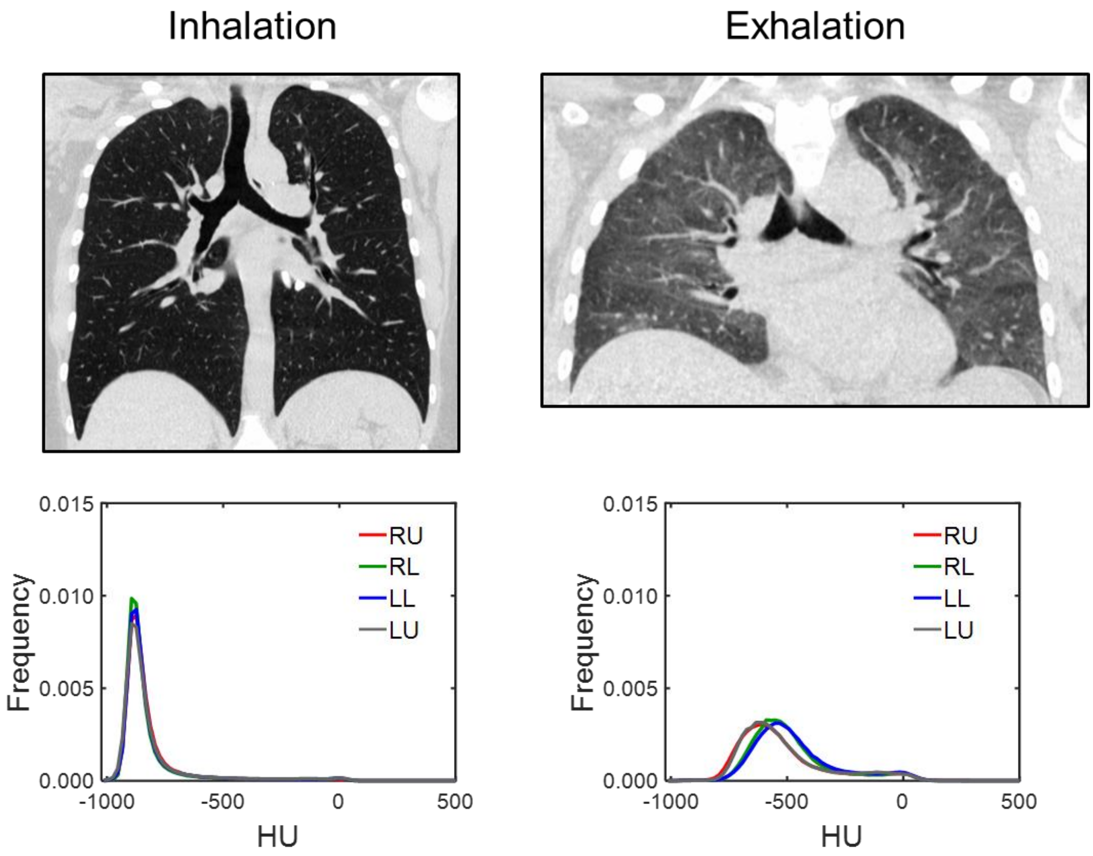

Figure S2

High-resolution computed tomography images and Hounsfield unit (HU) histograms of healthy subject H2. RU denotes the right upper and right middle lobes combined. RL, LL, and LU denote the right lower, left lower, and left upper lobes, respectively.

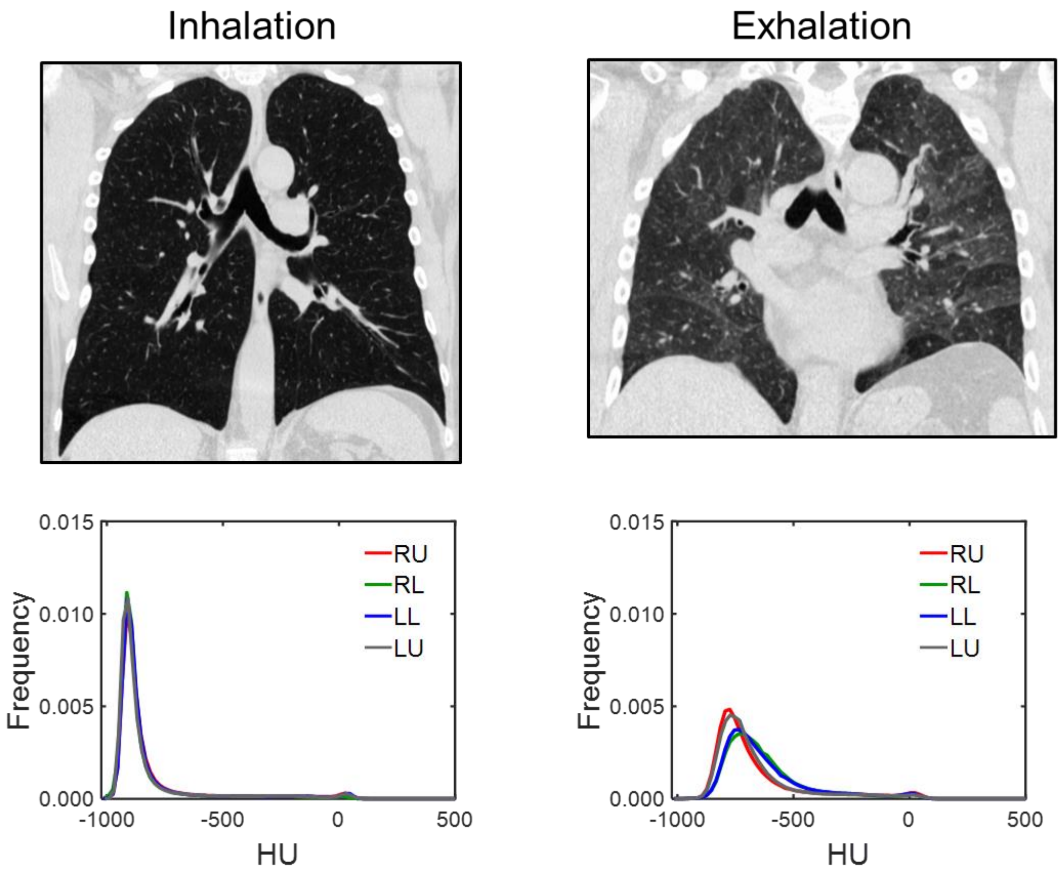

Figure S3

High-resolution computed tomography images and Hounsfield unit (HU) histograms of healthy subject H3. RU denotes the right upper and right middle lobes combined. RL, LL, and LU denote the right lower, left lower, and left upper lobes, respectively.

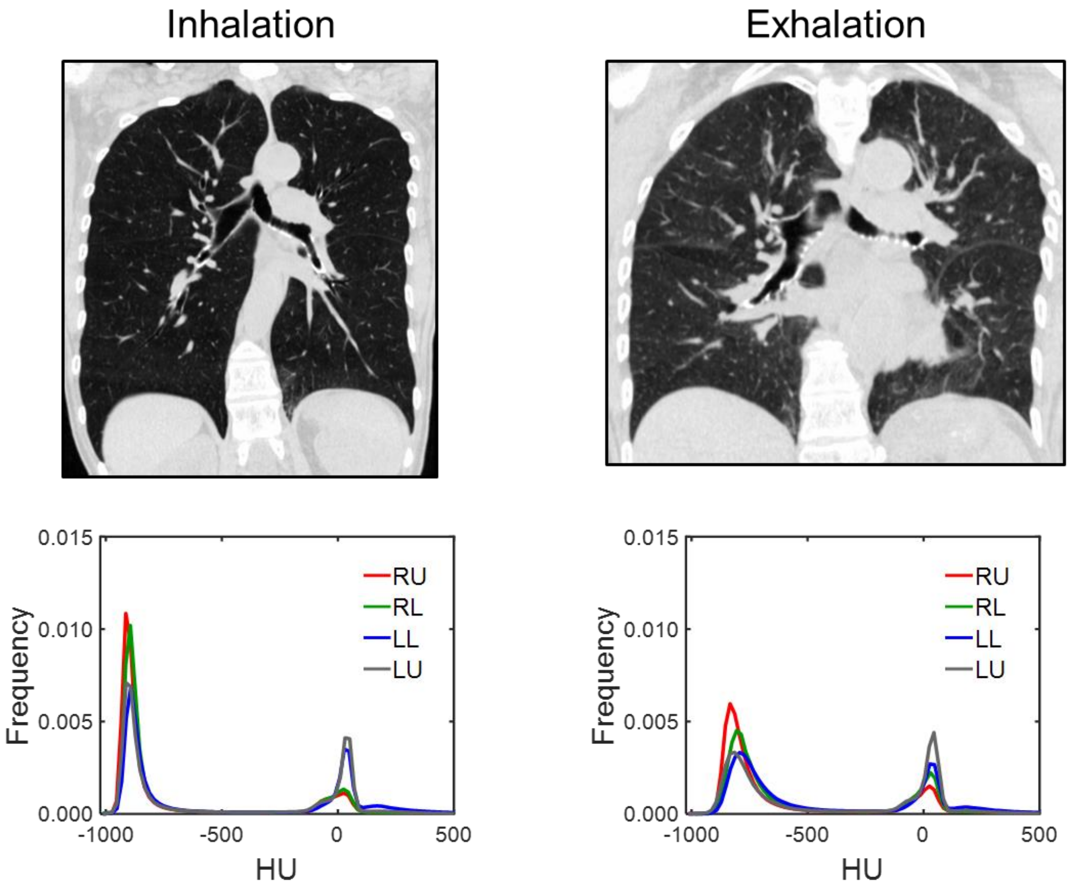

Figure S4

High-resolution computed tomography images and Hounsfield unit (HU) histograms of healthy subject H4. RU denotes the right upper and right middle lobes combined. RL, LL, and LU denote the right lower, left lower, and left upper lobes, respectively.

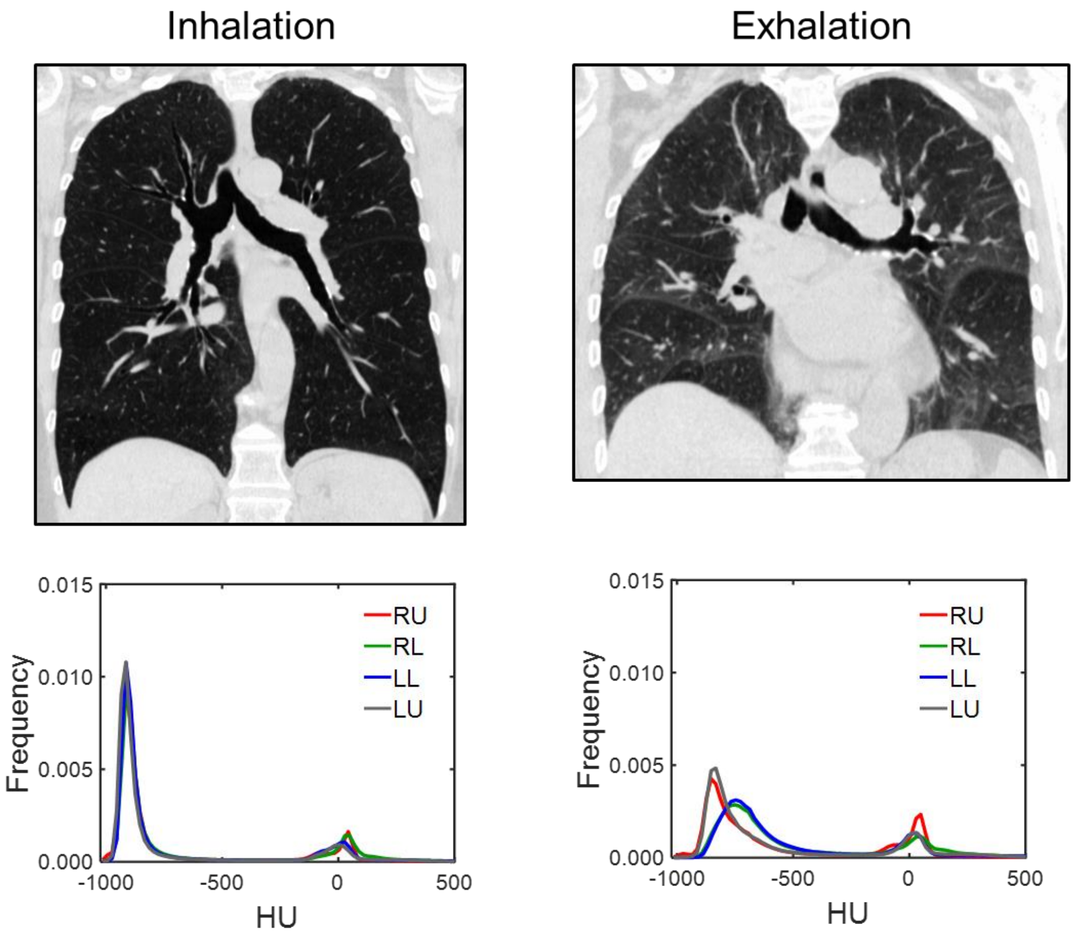

Figure S5

High-resolution computed tomography images and Hounsfield unit (HU) histograms of healthy subject H5. RU denotes the right upper and right middle lobes combined. RL, LL, and LU denote the right lower, left lower, and left upper lobes, respectively.

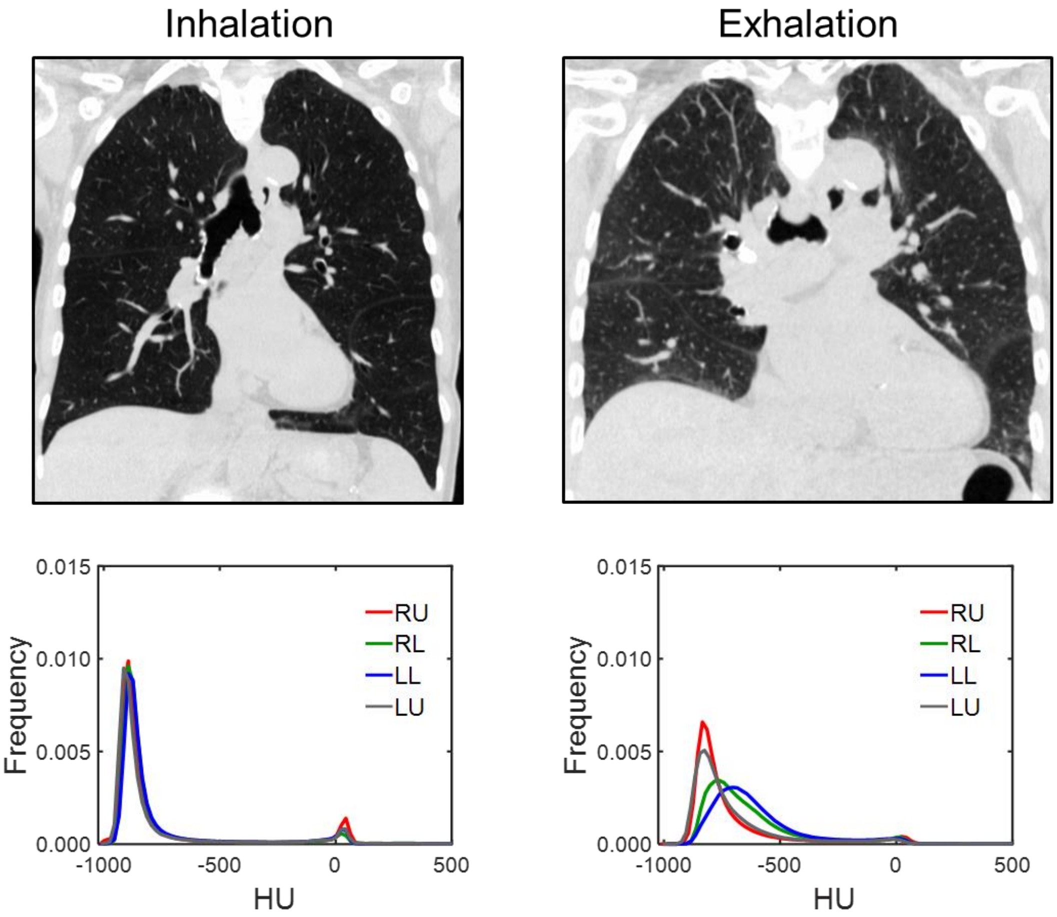

Figure S6

High-resolution computed tomography images and Hounsfield unit (HU) histograms of healthy subject H6. RU denotes the right upper and right middle lobes combined. RL, LL, and LU denote the right lower, left lower, and left upper lobes, respectively.

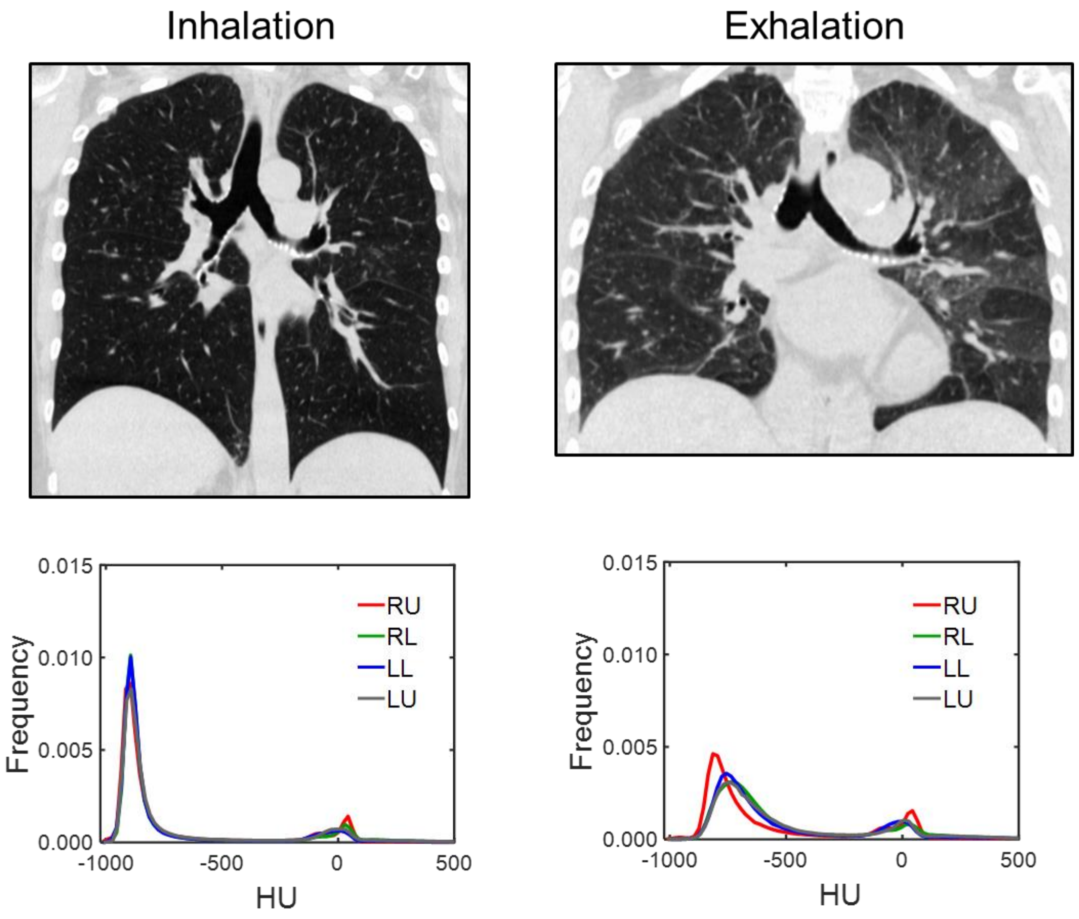

Figure S7

High-resolution computed tomography images and Hounsfield unit (HU) histograms of healthy subject H7. RU denotes the right upper and right middle lobes combined. RL, LL, and LU denote the right lower, left lower, and left upper lobes, respectively.

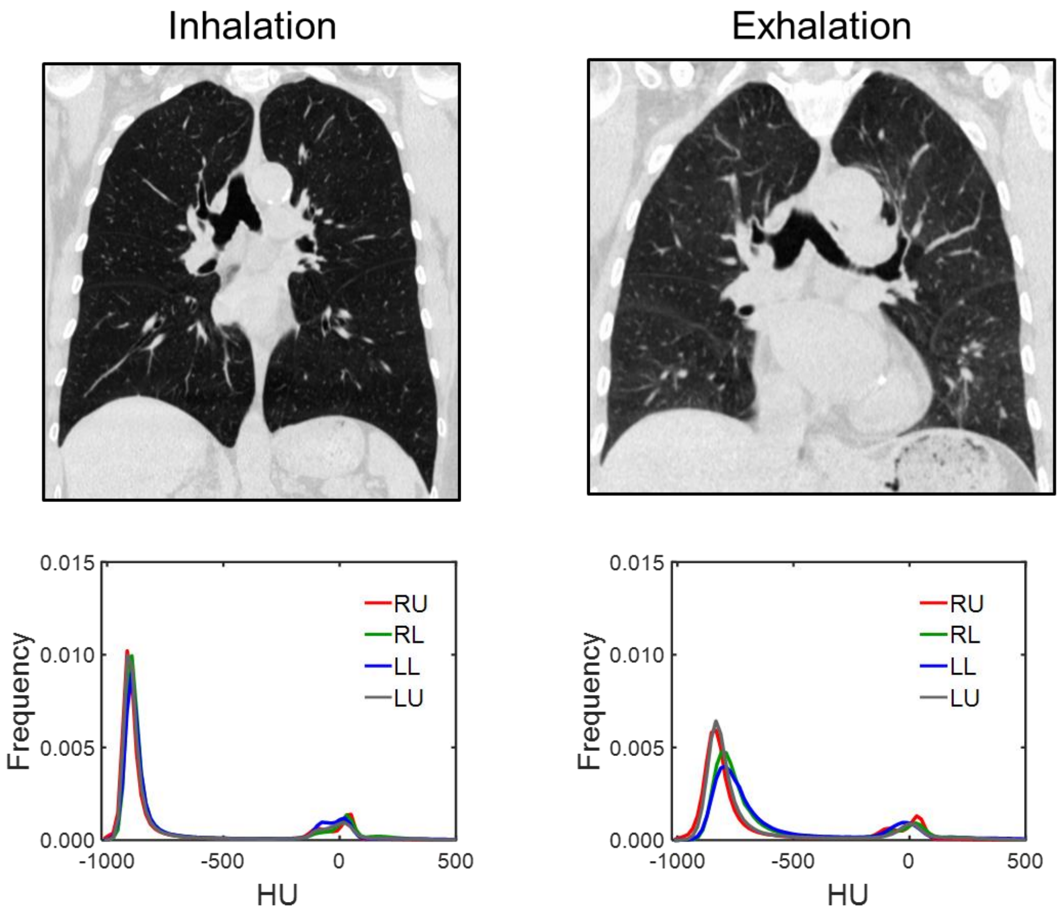

Figure S8

High-resolution computed tomography images and Hounsfield unit (HU) histograms of healthy subject H8. RU denotes the right upper and right middle lobes combined. RL, LL, and LU denote the right lower, left lower, and left upper lobes, respectively.

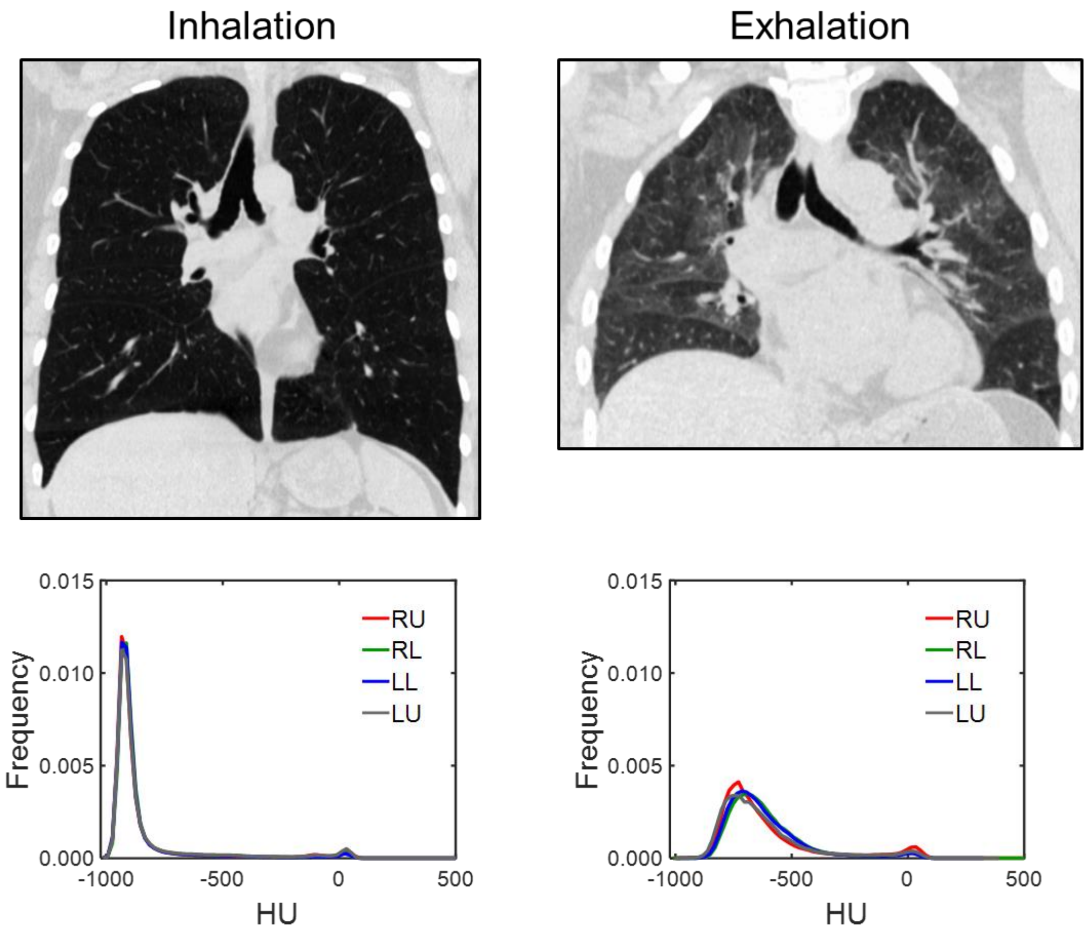

Figure S9

High-resolution computed tomography images and Hounsfield unit (HU) histograms of healthy subject H9. RU denotes the right upper and right middle lobes combined. RL, LL, and LU denote the right lower, left lower, and left upper lobes, respectively.

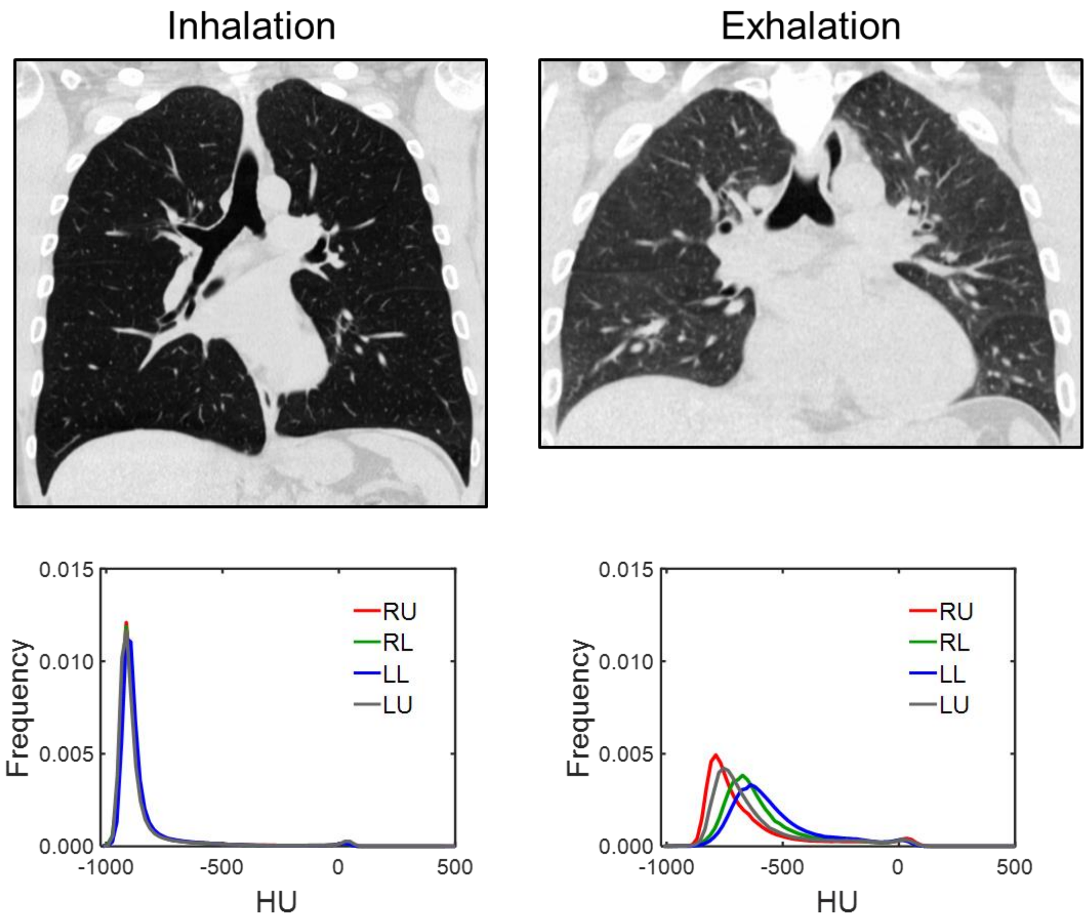

Figure S10

High-resolution computed tomography images and Hounsfield unit (HU) histograms of healthy subject H10. RU denotes the right upper and right middle lobes combined. RL, LL, and LU denote the right lower, left lower, and left upper lobes, respectively.

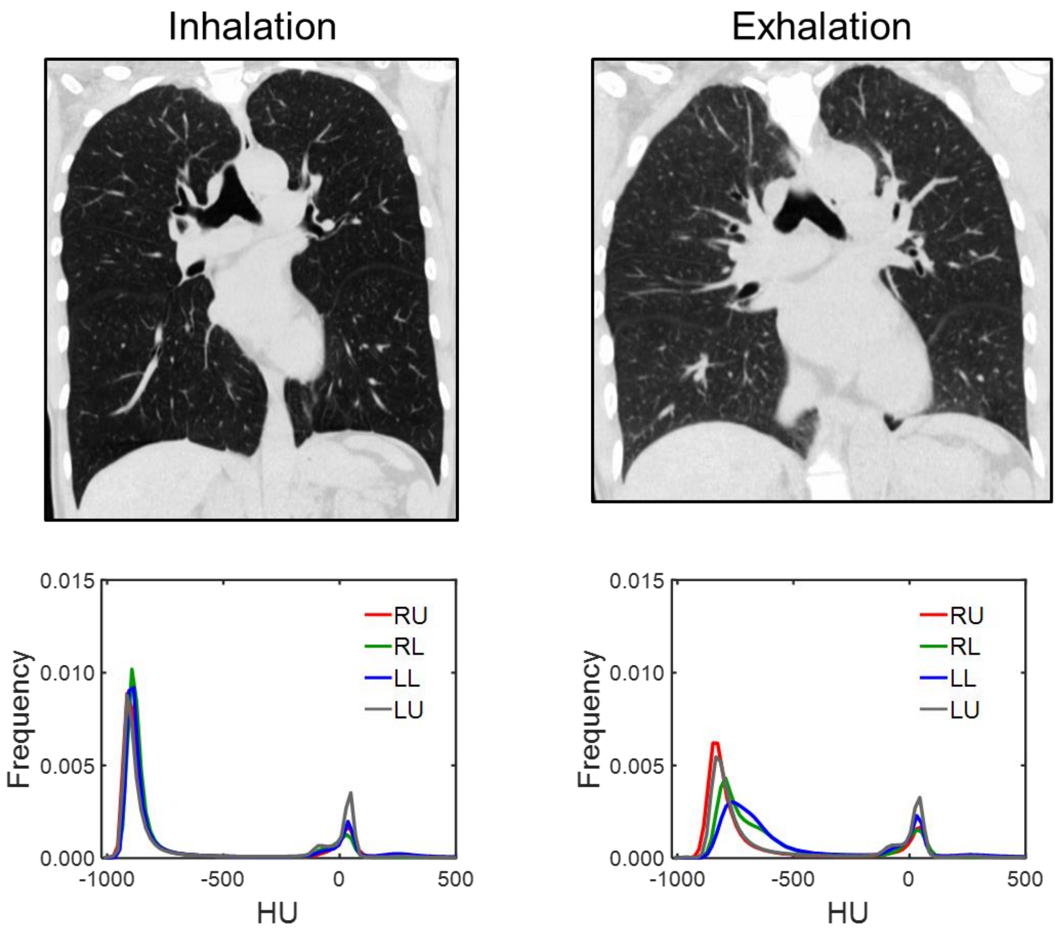

Figure S11

High-resolution computed tomography images and Hounsfield unit (HU) histograms of healthy subject H11. RU denotes the right upper and right middle lobes combined. RL, LL, and LU denote the right lower, left lower, and left upper lobes, respectively.

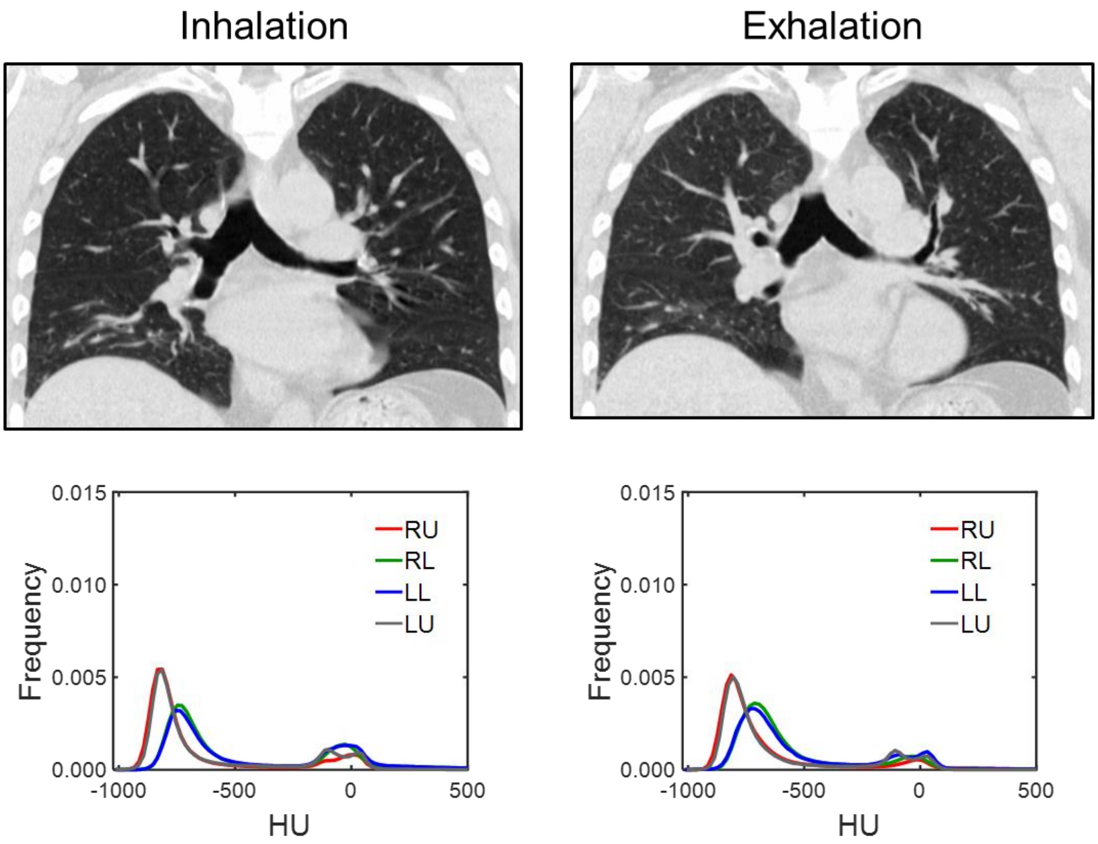

Figure S12

High-resolution computed tomography images and Hounsfield unit (HU) histograms of healthy subject H12. RU denotes the right upper and right middle lobes combined. RL, LL, and LU denote the right lower, left lower, and left upper lobes, respectively.

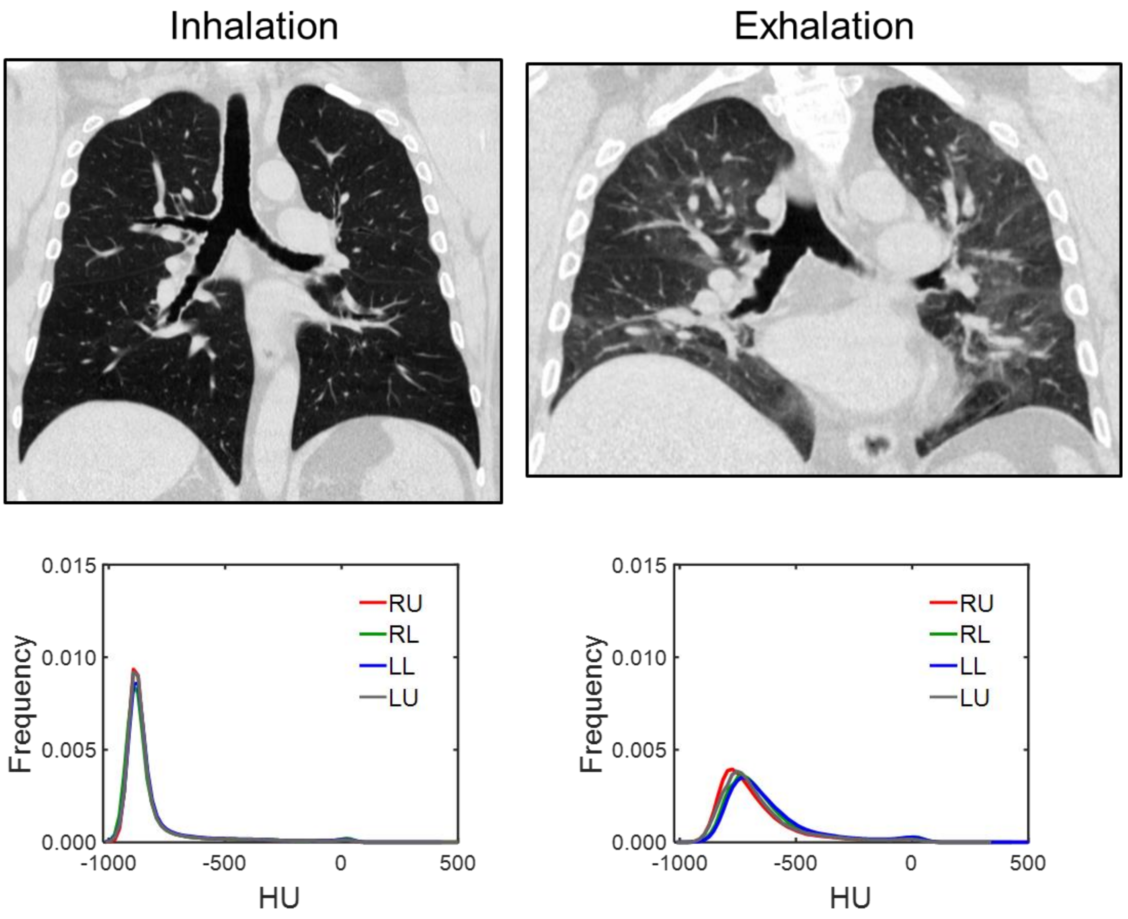

Figure S13

High-resolution computed tomography images and Hounsfield unit (HU) histograms of healthy subject H13. RU denotes the right upper and right middle lobes combined. RL, LL, and LU denote the right lower, left lower, and left upper lobes, respectively.

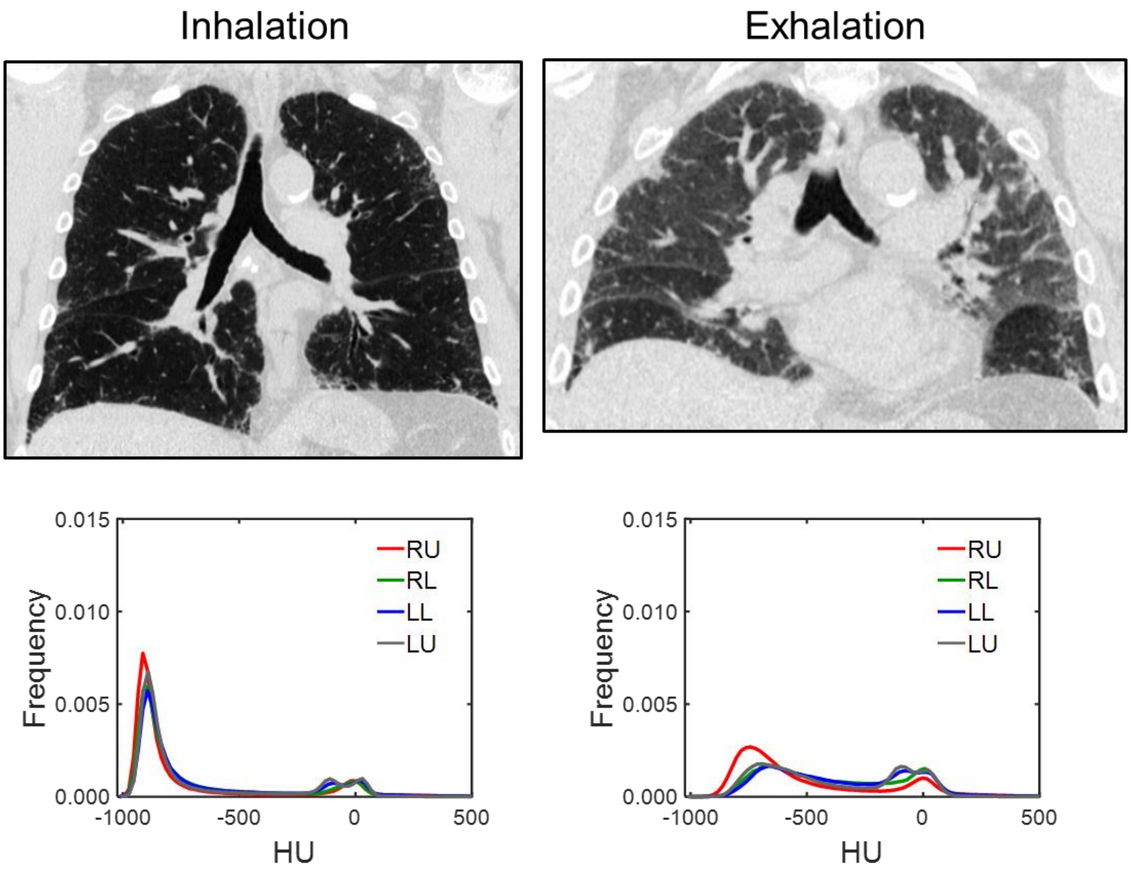

Figure S14

High-resolution computed tomography images and Hounsfield unit (HU) histograms of idiopathic pulmonary fibrosis subject D1. RU denotes the right upper and right middle lobes combined. RL, LL, and LU denote the right lower, left lower, and left upper lobes, respectively.

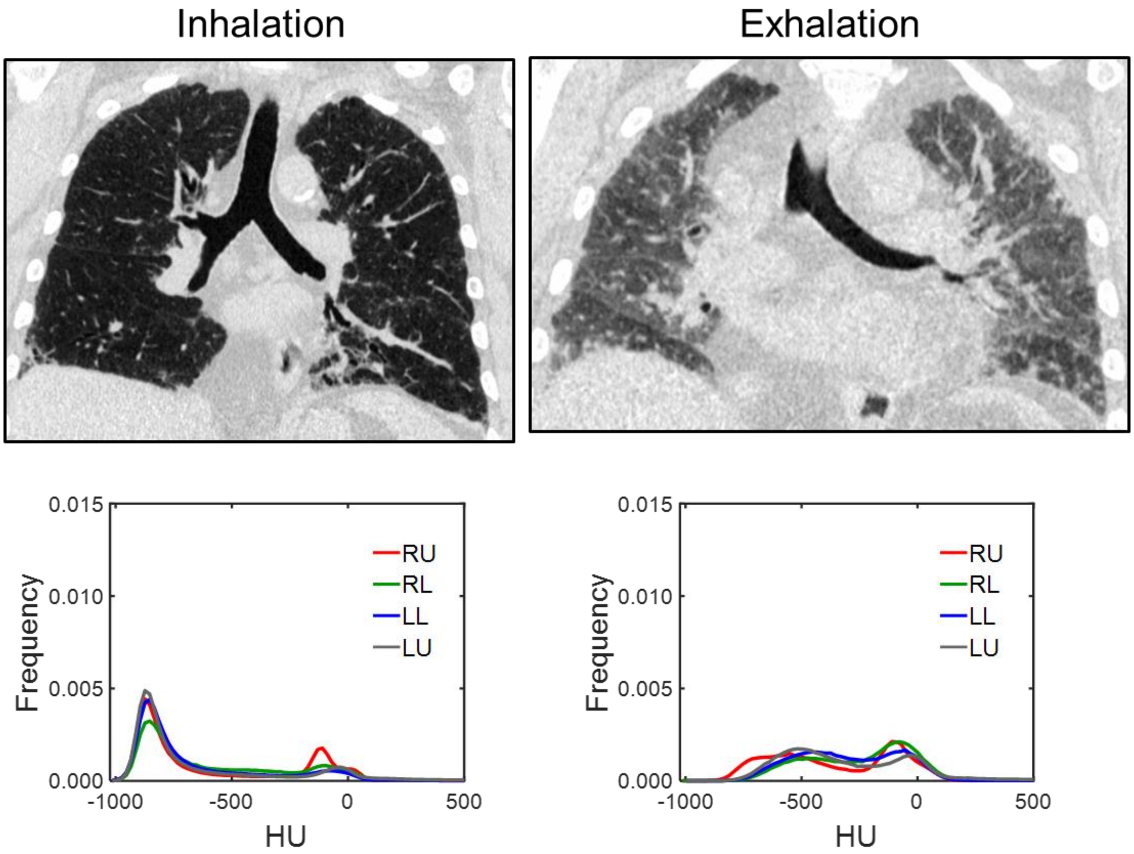

Figure S15

High-resolution computed tomography images and Hounsfield unit (HU) histograms of idiopathic pulmonary fibrosis subject D2. RU denotes the right upper and right middle lobes combined. RL, LL, and LU denote the right lower, left lower, and left upper lobes, respectively.

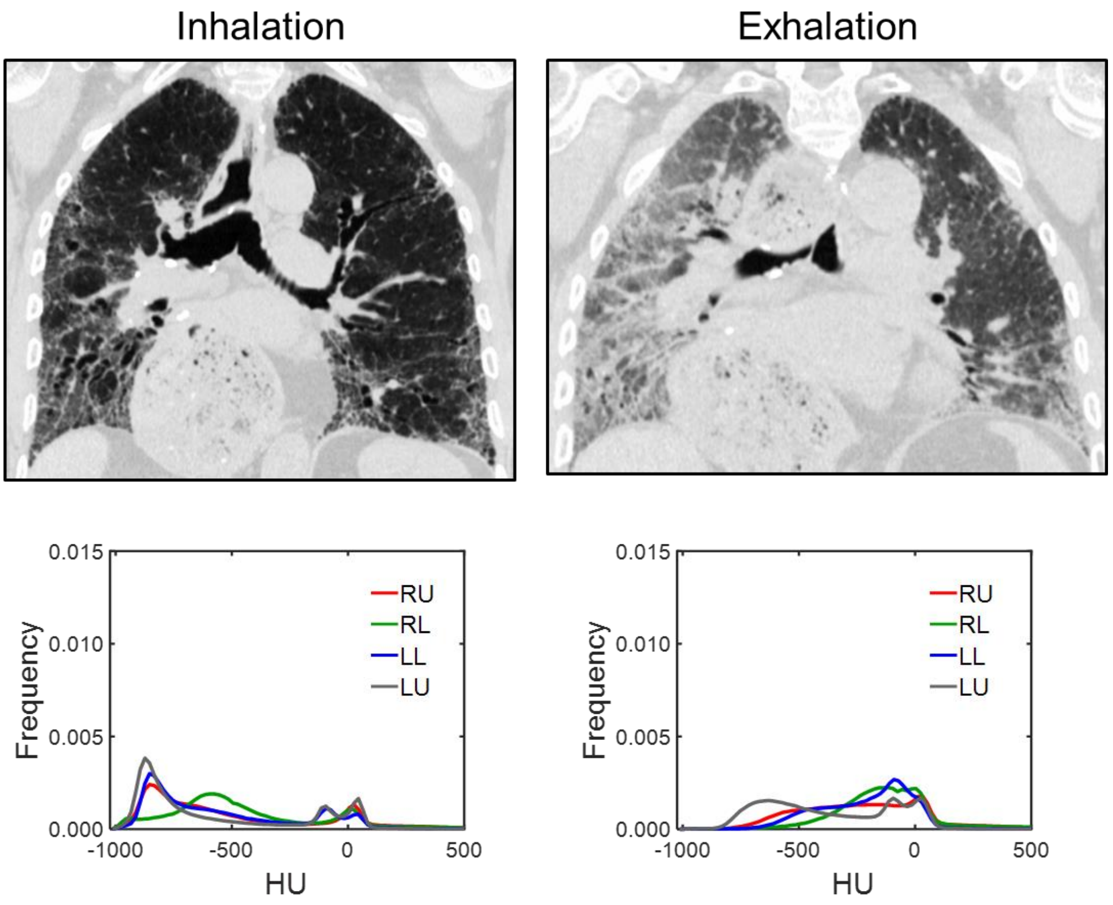

Figure S16

High-resolution computed tomography images and Hounsfield unit (HU) histograms of idiopathic pulmonary fibrosis subject D3. RU denotes the right upper and right middle lobes combined. RL, LL, and LU denote the right lower, left lower, and left upper lobes, respectively.

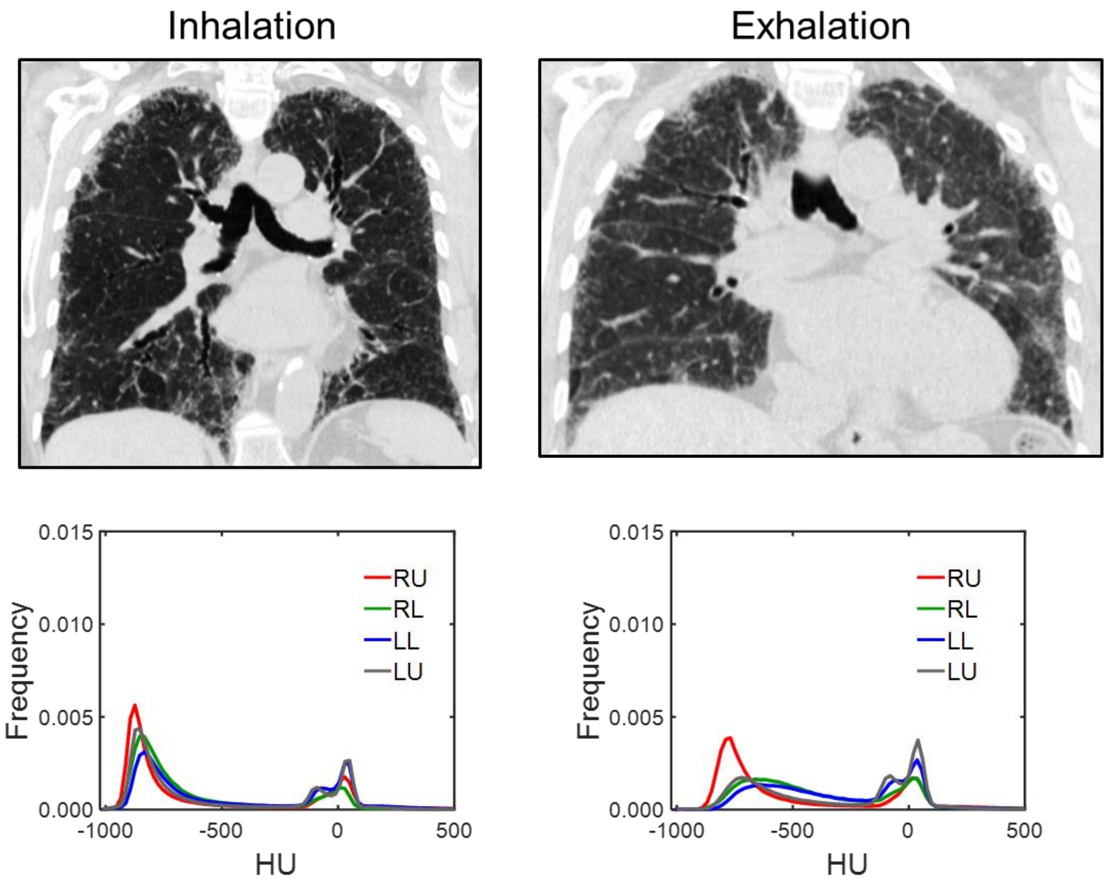

Figure S17

High-resolution computed tomography images and Hounsfield unit (HU) histograms of idiopathic pulmonary fibrosis subject D4. RU denotes the right upper and right middle lobes combined. RL, LL, and LU denote the right lower, left lower, and left upper lobes, respectively.

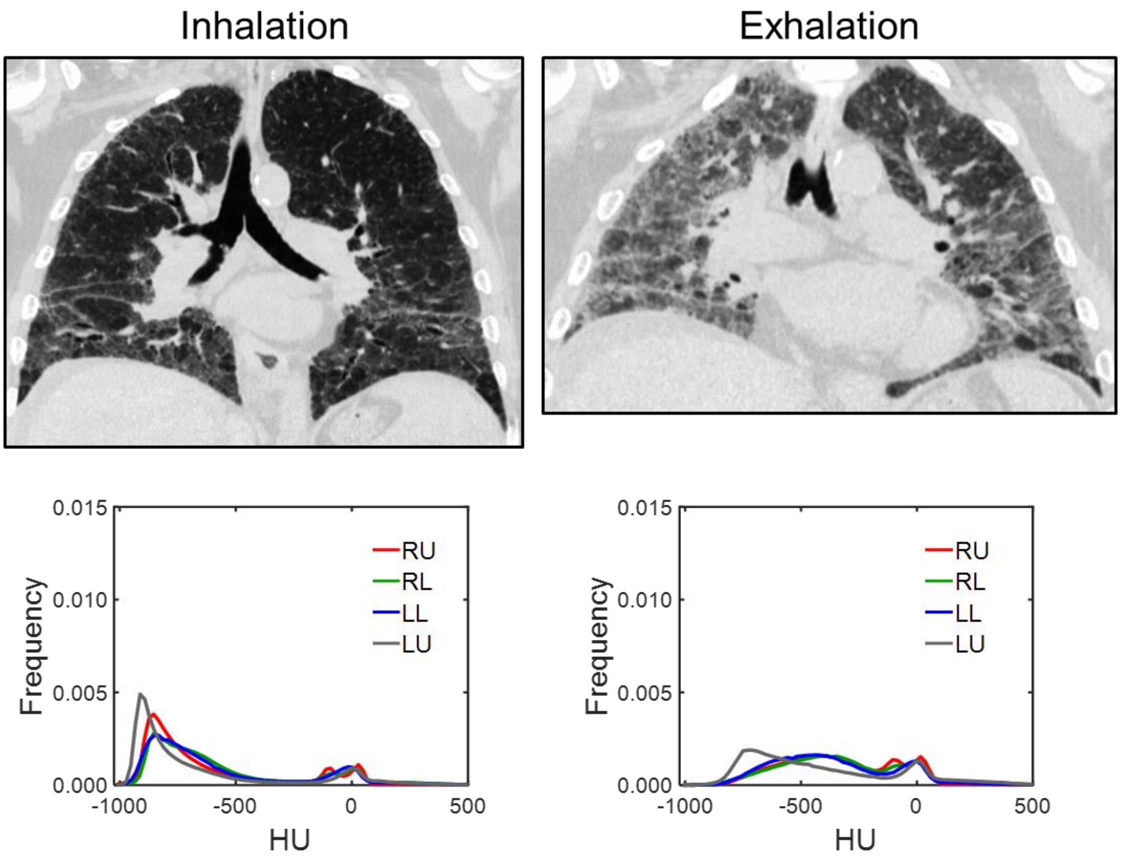

Figure S18

High-resolution computed tomography images and Hounsfield unit (HU) histograms of idiopathic pulmonary fibrosis subject D5. RU denotes the right upper and right middle lobes combined. RL, LL, and LU denote the right lower, left lower, and left upper lobes, respectively.

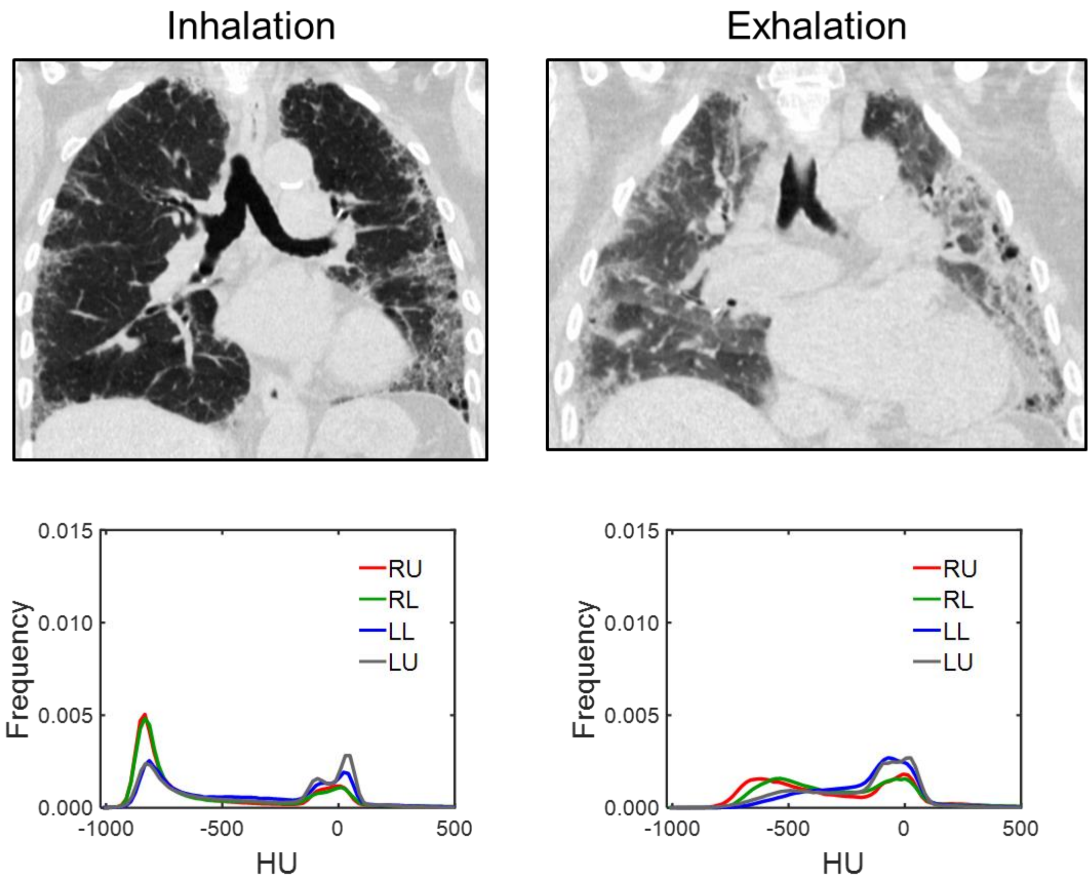

Figure S19

High-resolution computed tomography images and Hounsfield unit (HU) histograms of idiopathic pulmonary fibrosis subject D6. RU denotes the right upper and right middle lobes combined. RL, LL, and LU denote the right lower, left lower, and left upper lobes, respectively.

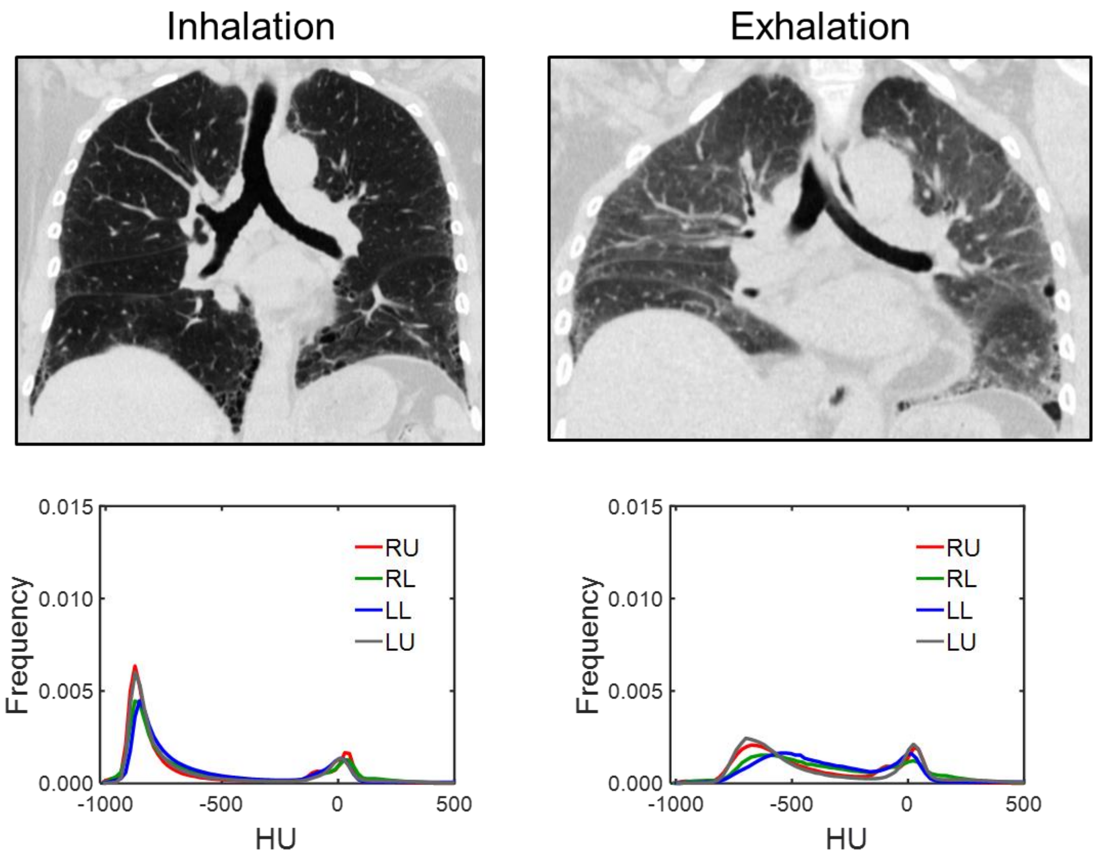

Figure S20

High-resolution computed tomography images and Hounsfield unit (HU) histograms of idiopathic pulmonary fibrosis subject D7. RU denotes the right upper and right middle lobes combined. RL, LL, and LU denote the right lower, left lower, and left upper lobes, respectively.

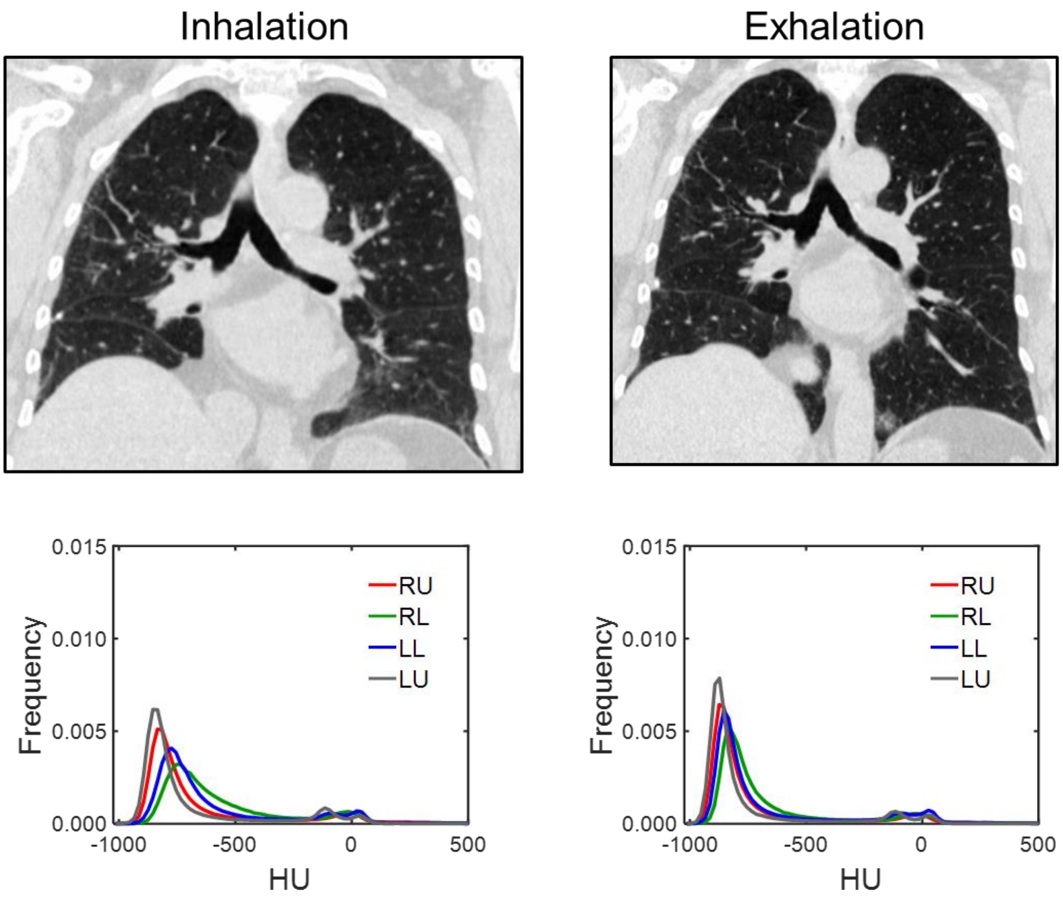

Figure S21

High-resolution computed tomography images and Hounsfield unit (HU) histograms of idiopathic pulmonary fibrosis subject D8. RU denotes the right upper and right middle lobes combined. RL, LL, and LU denote the right lower, left lower, and left upper lobes, respectively.

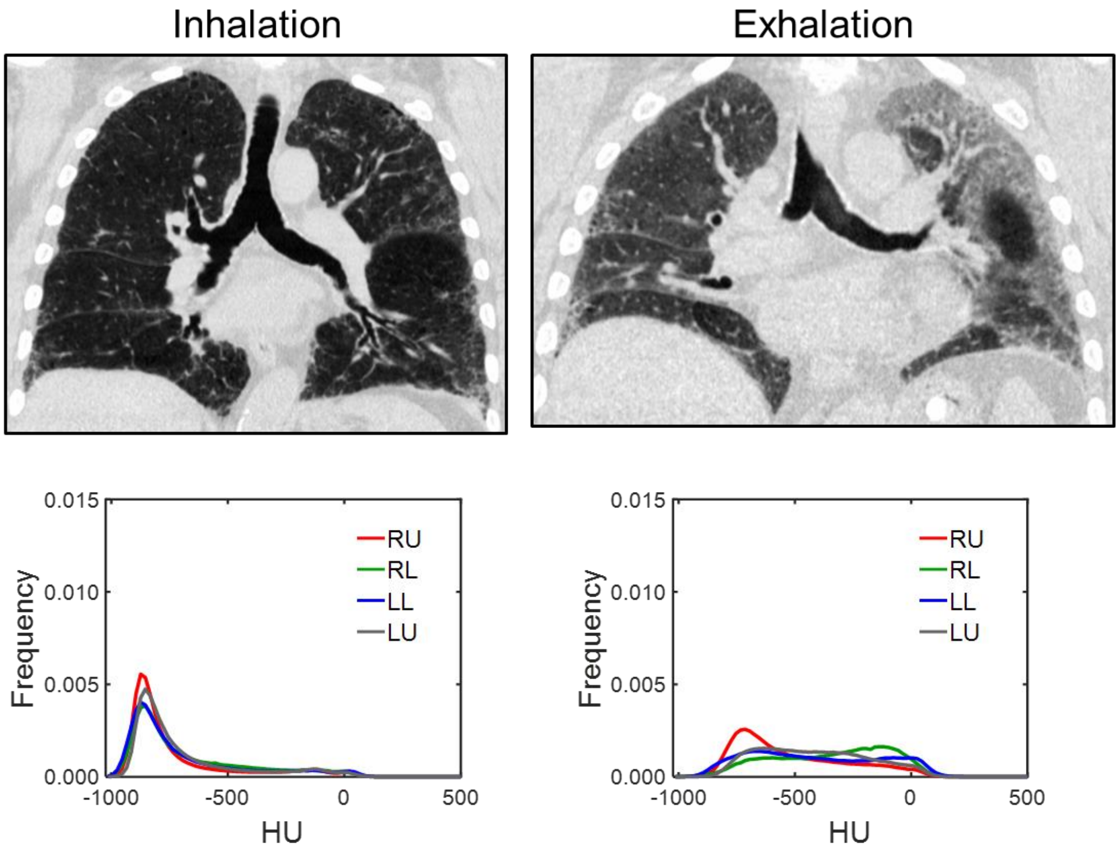

Figure S22

High-resolution computed tomography images and Hounsfield unit (HU) histograms of idiopathic pulmonary fibrosis subject D9. RU denotes the right upper and right middle lobes combined. RL, LL, and LU denote the right lower, left lower, and left upper lobes, respectively.

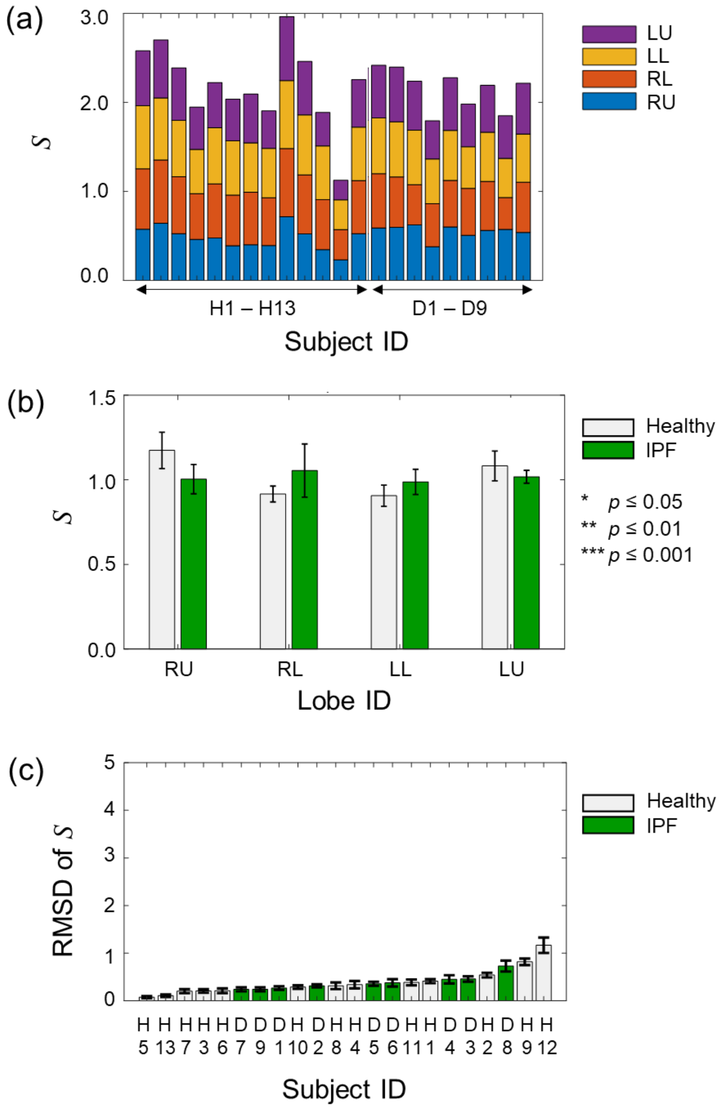

Figure S23

The strain of the lung lobes (S; See Eq. 4 in the Methods Section), defined as the difference in lobe volume between the expanded and contracted states normalized by the lobe volume in the expanded state, for the healthy (N = 13) and idiopathic pulmonary fibrosis (IPF) subjects (N = 9). (a) S values of healthy (H1–H13) and IPF subjects (D1–D9). (b) Mean values of S for the healthy and IPF groups. The error bars denote one standard deviation and the asterisks indicate different levels of statistical significance for differences in the mean values between the two groups. (c) Individual root-mean-squared difference (RMSD) values of S (See Eq. 5 in the Methods Section). The error bars denote one standard deviation computed from 13 iterative leave-one-out validations (See the Methods Section). RU denotes the right upper and right middle lobes combined. RL, LL, and LU denote the right lower, left lower, and left upper lobes, respectively.
